# Albumosomes formed by cytoplasmic pre-folding albumin maintain mitochondrial homeostasis and inhibit nonalcoholic fatty liver disease

**DOI:** 10.1038/s41392-023-01437-0

**Published:** 2023-06-16

**Authors:** Boyuan Ma, Anji Ju, Shaosen Zhang, Qi An, Siran Xu, Jie Liu, Li Yu, Yan Fu, Yongzhang Luo

**Affiliations:** 1 School of Life Sciences, Tsinghua University, 100084 Beijing, China; 2 The National Engineering Research Center for Protein Technology, Tsinghua University, 100084 Beijing, China; 3 Beijing Key Laboratory for Protein Therapeutics, Tsinghua University, 100084 Beijing, China; 4 State Key Laboratory of Membrane Biology, Tsinghua University-Peking University Joint Centre for Life Sciences, Beijing Frontier Research Center for Biological Structure, School of Life Sciences, Tsinghua University, 100084 Beijing, China; 5 Present Address: Department of Etiology and Carcinogenesis, National Cancer Center/Cancer Hospital, Chinese Academy of Medical Sciences and Peking Union Medical College, 100021 Beijing, China; 6 Present Address: Immunogenetics Laboratory, Shenzhen Blood Center, 518025 Shenzhen, Guangdong China

**Keywords:** Cell biology, Metabolic disorders

## Abstract

Hepatic mitochondrial dysfunction contributes to the progression of nonalcoholic fatty liver disease (NAFLD). However, the factors that maintain mitochondrial homeostasis, especially in hepatocytes, are largely unknown. Hepatocytes synthesize various high-level plasma proteins, among which albumin is most abundant. In this study, we found that pre-folding albumin in the cytoplasm is completely different from folded albumin in the serum. Mechanistically, endogenous pre-folding albumin undergoes phase transition in the cytoplasm to form a shell-like spherical structure, which we call the “albumosome”. Albumosomes interact with and trap pre-folding carnitine palmitoyltransferase 2 (CPT2) in the cytoplasm. Albumosomes control the excessive sorting of CPT2 to the mitochondria under high-fat-diet-induced stress conditions; in this way, albumosomes maintain mitochondrial homeostasis from exhaustion. Physiologically, albumosomes accumulate in hepatocytes during murine aging and protect the livers of aged mice from mitochondrial damage and fat deposition. Morphologically, mature albumosomes have a mean diameter of 4μm and are surrounded by heat shock protein Hsp90 and Hsp70 family proteins, forming a larger shell. The Hsp90 inhibitor 17-AAG promotes hepatic albumosomal accumulation in vitro and in vivo, through which suppressing the progression of NAFLD in mice.

## Introduction

Nonalcoholic fatty liver disease (NAFLD) has recently become the most common chronic liver disease and the fastest growing cause of hepatocellular carcinoma in many regions of the world, including the USA and parts of Europe.^1–3^ Pathologically, excessive fatty acid intake due to the consumption of a high-fat-diet (HFD) leads to the storage of lipids in hepatocytes,^4^ which stimulates fatty acid oxidation, the tricarboxylic acid (TCA) cycle, and oxidative phosphorylation in mitochondria.^5–8^ Nicotinamide adenine dinucleotide hydrogen (NADH) and flavin adenine dinucleotide hydrogen_2_ (FADH_2_), which are generated by these processes, donate electrons to the electron transport chain (ETC) for adenosine triphosphate (ATP) synthesis.^9,10^ However, a small fraction of high-energy electrons leaks from the ETC and directly reacts with oxygen to generate superoxide radicals, which are the main source of reactive oxidative species (ROS) inside mitochondria.^9–13^ Under metabolic stress conditions, the production of ROS inside the mitochondria increases, resulting in a ROS level exceeding neutralizing ability of the antioxidative system.^9,12–15^ ROS destroy the mitochondrial membrane and mitochondrial DNA, and also induce hepatic inflammation *via* the nuclear factor-κB (NF-κB) and nucleotide-binding oligomerization domain-like receptor family pyrin domain-containing 3 (NLRP3) pathways.^14,15^ Therefore, although mitochondria can maintain a balance between ROS generation and antioxidation under normal conditions, they are damaged by excessive metabolic pressure.^5–9^ Moreover, impaired mitochondrial function decreases the rate of fatty acid metabolism and exacerbates lipid accumulation.^11,12^ Thus, severe nutrient stress conditions due to the consumption of an HFD result in a vicious cycle between hepatic lipid accumulation and mitochondrial exhaustion, and the consequence is hepatocyte death and lipotoxic liver injury.^9^ Hence, the maintenance of hepatic mitochondrial homeostasis under nutrient stress conditions is essential for maintaining metabolic balance and treating NAFLD.^16–18^ However, the factors that maintain mitochondrial homeostasis during NAFLD, especially for hepatocytes, are largely unknown.^1–3^

One of the unique characteristics of hepatocytes, which function as protein secretion factories, is that they synthesize high levels of various basic blood proteins such as albumin, transferrin, and lipoproteins.^19^ Thus, due to their active anabolic and metabolic states, hepatocytes have a large size ranging from 20 to 30 μm, and are rich in different organelles.^20–22^ It has been reported that the contacts between mitochondria and other organelles such as the endoplasmic reticulum (ER) and lipid droplets (LDs) directly contribute to cellular lipid metabolism and energy homeostasis.^23,24^ However, not all subcellular structures involved in mitochondrial homeostasis have been clearly identified, particularly hepatocytes-specific organelles.

Recently, accumulating studies have revealed numerous membrane-less organelles that are formed *via* protein phase separation and transition.^25,26^ Biomacromolecules phase separate or transit to form subcellular high-concentrated complexes, enabling increased rates of biochemical reactions (transcription, signal transduction, autophagy, LD fusion, etc.)^27–38^ or storing relevant RNA and proteins.^39–42^ Moreover, it has also been reported that phase separation into quinary assemblies is a survival strategy for organisms under stressful conditions.^43,44^ Thus, these organelles often acquire new functions in unexpected ways, which inspired us to explore novel organelles within hepatocytes that provide protection under lipotoxic pressure. Since the intracellular concentration of a protein is one of the key prerequisites for phase separation and transition,^25,26^ we focused on albumin, which is expressed at the highest level among all hepatic-specific proteins.

In the current study, we find that intracellular pre-folding albumin undergoes a phase transition to form a spherical shell-like structure in the cytoplasm, which we name “albumosome”. Albumosomes accumulate in hepatocytes along with the physiological aging of wild-type (WT) mice, and the livers of albumin-knockout (AKO) mice, which lack albumosomes, have more severe fat deposition and damaged mitochondria than those of WT mice of the same age. Mechanistically, albumosomes bind to and trap pre-folding carnitine palmitoyltransferase 2 (CPT2) in the cytoplasm and control its sorting to mitochondria. Through the interaction, the mitochondrial respiratory rate is regulated, and the excessive nutrient stress in mitochondria is partially relieved. Morphologically, members of the Hsp90 family (Hsp90α and Hsp90β) and Hsp70 family (Hsp70 and Hsc70) surround the outer surface of albumosomes as a larger shell. The Hsp90 inhibitor 17-AAG promotes the accumulation of albumosomes in vitro and in vivo, thus suppresses the progression of NAFLD and obesity in HFD-fed mice. These findings reveal that albumosomes have mitochondrial protective functions in hepatocytes.

## Results

### Albumin knockout triggers NAFLD and obesity progression during the aging process

**Fig. 1:**
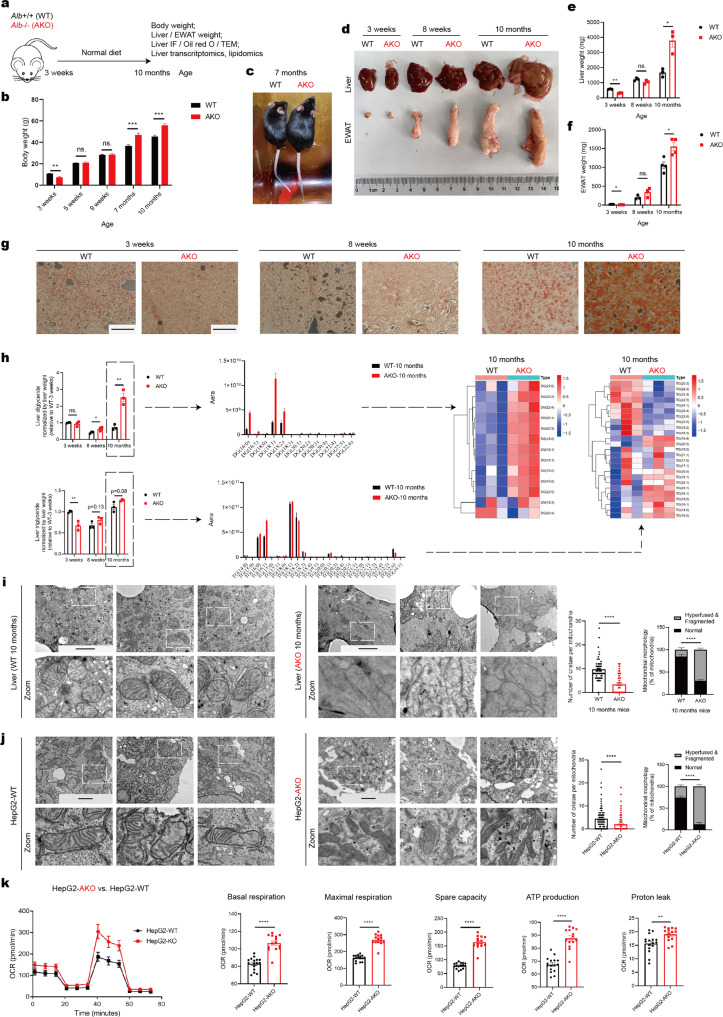
Albumin knockout promotes fatty liver disease progression, obesity, and mitochondrial damage during the aging process. **a** Experimental design model. WT and AKO mice were fed with the normal diet for 10 months (*n* = 3–10). **b** Body weights of WT and AKO mice at the age of 3 weeks, 5 weeks, 9 weeks, 7 months, and 10 months (*n* = 3–10). **c** Representative images of WT and AKO mice at the age of 7 months. **d** WT and AKO mice at the age of 3 weeks, 8 weeks, and 10 months were euthanized. Their livers and EWAT were obtained and weighed. Representative images of livers and EWAT (*n* = 3–4). **e**, **f** Weights of the livers (**e**) and EWAT (**f**) from the mice in **d** (*n* = 3–4). **g** Liver frozen sections from the mice at 3 weeks, 8 weeks, and 10 months of age were stained by Oil Red O. Representative images of the sections were shown (*n* = 3–4). Scale bars: 1 mm. **h** Lipid composition of the livers from WT and AKO mice at 3 weeks, 8 weeks, and 10 months of age were analyzed by lipidomics. Total amount of major lipids diglyceride and triglyceride of 10-month-old livers were shown. Fatty acids composition of diglyceride and triglyceride in 10-month-old livers were shown. Heatmap of the relative levels of all fatty acids in diglyceride and triglyceride of 10-month-old livers (*n* = 3). **i** TEM of liver sections of 10-month-old WT and AKO mice. The number of cristae per mitochondria and the hyperfused and fragmented level of mitochondria were analyzed. Representative images were shown. Scale bars: 2 μm. **j** TEM of HepG2-WT and HepG2-AKO. The number of cristae per mitochondria and the hyperfused and fragmented level of mitochondria were analyzed. Representative images were shown. Scale bars: 2 μm. **k** Seahorse mitochondrial respiratory assay of HepG2-WT and HepG2-AKO. Basal respiration, ATP production, proton leak, maximal respiration and spare capacity were analyzed. Data are analyzed by unpaired two-tailed Student’s *t* test and represented as mean ± SEM. ns. represents no significance. *p < 0.05, **p < 0.01, ***p < 0.001, ****p < 0.0001

We observed the phenotypic changes in *Alb*−/− (AKO) mice from the age of 3 weeks to 10 months, and used *Alb*+/+ (WT) mice as a control (Fig. 1a). Firstly, the body weight of the AKO mice changed more dramatically with aging (Fig. 1b). During the juvenile period (3 weeks of age), the AKO mice weighed less than the WT mice (Fig. 1b). By the time of sexual maturity (5–9 weeks of age), there was no significant difference between the two groups (Fig. 1b). However, as aging progressed, the AKO mice became significantly heavier than the WT mice after 7 months of age (Fig. 1b, c).

Thus, we selected three representative ages (3 weeks, 8 weeks, and 10 months) and assessed the differences in livers and epididymis white adipose tissues (EWAT) between the WT and AKO mice. Morphologically, albumin knockout led to hepatic hypertrophy, yellowing and hardening at 10 months of age, which was consistent with the appearance of fatty liver reported in the literature^45,46^ (Fig. 1d). In terms of weight, the livers and EWAT of the AKO mice were significantly heavier than those of the WT mice especially at 10 months of age (Fig. 1e, f). Moreover, liver oil red O staining in each group showed that LDs were larger and more abundant in the 10-month-old AKO mice than in the WT mice (Fig. 1g). However, at an earlier age, the livers and EWAT of the AKO mice had weights that were similar to (8 weeks) or even lighter than (3 weeks) those of the WT mice (Fig. 1d–f); these results indicate that the function of albumin in maintaining lipid homeostasis is mediated not only by albumin itself but also by the aging process.

We further explored the lipid composition of the livers in mice of each group. By lipidomic analysis, we found that the total levels of diglycerides (DG) in the livers of the AKO mice were more than twice as high as those of the WT mice; specifically, the contents of the major fatty acids DG (18:1), DG (18:2) and DG (16:0) were all significantly higher than those of the WT mice (Fig. 1h). Although the total triglyceride (TG) levels of the AKO livers were not significantly higher than those of the WT livers at 10 months of age, the content of TG (16:1) was almost twice as high as that in the WT livers (Fig. 1h). For free fatty acids, the content of the major fatty acid 18:1 in the AKO livers was significantly higher than that in the WT livers (Supplementary Fig. S1a). These findings indicate that livers from aged AKO mice suffer from severe fat deposition.

Moreover, we used RNA-Seq to investigate the gene expression profiles of livers from the different groups and found that components of the fatty acid biosynthesis and fatty acid β-oxidation pathways were upregulated in 10-month-old AKO mice compared with WT mice (Supplementary Fig. S1b–d), which was consistent with hepatic hypertrophy and fat deposition observed in the livers (Fig. 1d, e, g, h); these results indicate that AKO livers stay at an exhausted and disordered state in terms of lipid metabolism.

### Albumin knockout leads to hepatic mitochondrial exhaustion in vivo and in vitro

Next, we explored the factors contribute to metabolic disorders in the livers of AKO mice. After analyzing the RNA-Seq results, we found that genes related to the mitochondrial respiratory chain were highly enriched in the livers of 10-month-old AKO mice, which suggests that the livers of AKO mice may have higher mitochondrial activity (Supplementary Fig. S1e, f). Ndufv1, Sdha, Uqcrc1 and Cox4 are essential subunit proteins in the Complex I, Complex II, Complex III, and Complex IV respectively. Therefore, we chose them to represent the expression levels and functions of these respiratory complexes. Since ATP8 is a vital subunit of the Complex V, and is also encoded by the mitochondrial DNA (mtDNA), we chose ATP8 to represent the changes in mtDNA and the Complex V. As a result, we found that the levels of the Complex I (Ndufv1) and the Complex II (Sdha) proteins significantly increased in AKO liver tissues (Supplementary Fig. S1g). However, the Complex III (Uqcrc1) had no significant change, and the Complex IV (Cox4) and the Complex V (ATP8) significantly decreased in AKO liver tissues (Supplementary Fig. S1g). These findings indicate that in aged mice, AKO triggered damage or dysfunction in the Complex IV and Complex V of hepatic mitochondria. Because continuous excessive respiration promotes ROS production and mitochondrial damage in NAFLD,^7,8,11^ we then explored the morphology of hepatic mitochondria in mice from each group by transmission electron microscopy (TEM). Ten-month-old WT mice had a normal hepatic mitochondrial morphology with clearly visible cristae (Fig. 1i). Notably, AKO hepatocytes showed a more crowded spatial arrangement of hepatic mitochondria and a significantly reduced number of cristae, indicating a damaged and abnormal state (Fig. 1i). It has been reported that some cellular stresses induce mitochondria into a hyperfused state (stress induced mitochondria hyperfusion, SIMH), in which the mitochondria contain condensed matrix and disorganized or disrupted cristae structures.^47–51^ SIMH promotes higher mitochondrial respiratory rate, but long-term SIMH causes mitochondrial fragmentation and dysfunction, and finally, apoptosis.^47,48^ Therefore, we analyzed the ultrastructure of mitochondria in each group in terms of the hyperfused level. The level of hyperfused and fragmented mitochondria in AKO livers was significantly higher than that in WT livers, indicating that AKO might trigger SIMH in mice livers (Fig. 1i). In addition, we isolated mouse primary hepatocytes from 8-week-old WT and AKO mice livers and analyzed their respiratory differences using the seahorse respiratory assay (Supplementary Fig. S2a). Consistent with the RNA-Seq results, AKO hepatocytes exhibited significantly higher level of respiratory rate than WT hepatocytes (Supplementary Fig. S2a), indicating excessive metabolic stresses in the AKO hepatocytes. This could explain that the Complex IV and Complex V of hepatic mitochondria exhibited dysfunction in AKO aged mice compared to WT aged mice (Supplementary Fig. S1g).

In addition, we generated an *ALB-*knockout HepG2 cell line (HepG2-AKO) to investigate its mitochondrial changes in vitro (Supplementary Fig. S2b). Using TEM, we found that the mitochondria in the HepG2-WT cells had normal morphology with a healthy membrane network and intact cristae (Fig. 1j). However, the mitochondrial cristae in the HepG2-AKO cells exhibited both significant shrinkage and decrease in number (Fig. 1j). Moreover, consistent with the ultrastructure of the mitochondria in mice livers, HepG2-AKO exhibited a significantly higher level of hyperfusion and fragmentation than HepG2-WT (Fig. 1j).

Based on the changes in mitochondrial ultrastructure due to deficiency of intracellular albumin, we investigated the mitochondrial respiratory rate of HepG2-WT and HepG2-AKO cells. Consistent with the primary mouse hepatocytes, HepG2-AKO cells exhibited higher respiratory level than the HepG2-WT cells (Fig. 1k), suggesting that albumin knockout trigger SIMH and mitochondrial exhaustion.^52^ Moreover, we analyzed the difference in metabolites between HepG2-WT and HepG2-AKO using the metabolomic assay. It was found that the key members in the TCA cycle such as citrate, *cis*-aconitate, succinate, fumarate, malate, and NADH were all significantly increased when albumin is knocked out, which indicates that intracellular albumin maintains the TCA cycle homeostasis in HepG2 (Supplementary Fig. S2c). Besides, we also found that some acylcarnitine such as myristoyl-carnitine-14, butyrylcarnitine, and L-acetylcarnitine were significantly increased in HepG2-AKO from the metabolomic results, which verifies that albumin knockout induces the fatty acid β-oxidation (Supplementary Fig. S2d), consistent with the RNA-Seq results in mouse livers (Supplementary Fig. S1c, d). These data indicate that hepatocytes without intracellular albumin are suffering from an excessive metabolic stress. Furthermore, we stained the cells with MitoSOX^53^ and found that the HepG2-AKO cells exhibited higher mitochondrial ROS levels than the HepG2-WT cells (Supplementary Fig. S2e). In summary, these findings demonstrate that deficiency of intracellular albumin triggers hepatic mitochondrial over-activation, and continuously, disorder and disruption.

### Intracellular albumin forms the albumosome, a novel structure in hepatocytes

How exactly does intracellular albumin remain and function in hepatocytes? The classical secretion theory states that as soon as synthesis occurs, signal peptides transport albumin to the ER for folding, and albumin is then secreted into the extracellular space via vesicles through the Golgi-apparatus.^54–56^ Therefore, no other form of intracellular albumin has been reported yet.

**Fig. 2:**
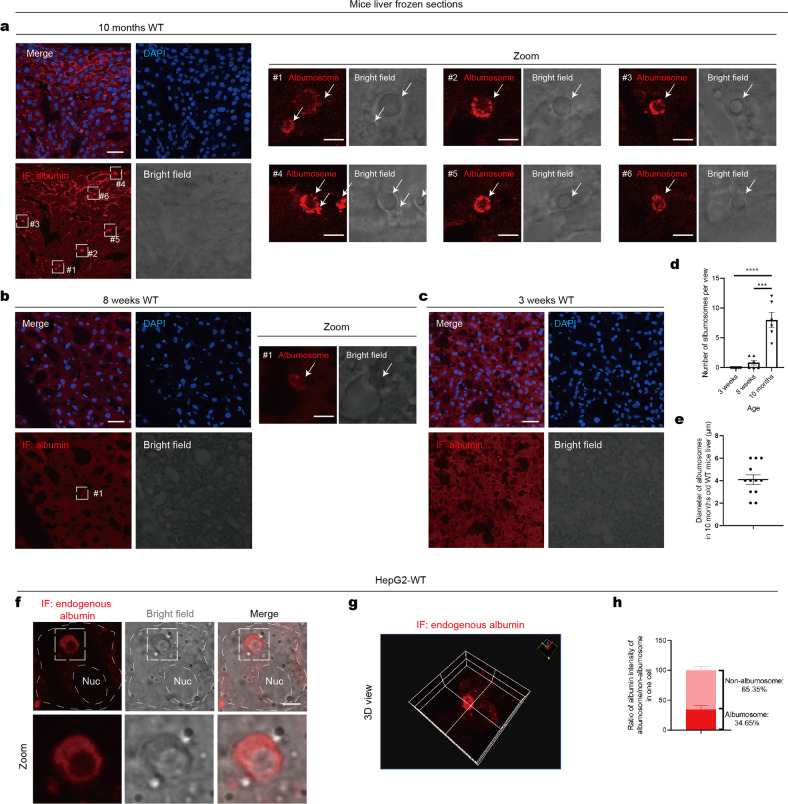
Intracellular albumin forms the albumosome, a novel structure in hepatocytes. **a**–**e** Liver sections of 10-month-old (**a**), 8-week-old (**b**), and 3-week-old (**c**) mice were used in IF. Representative images of maximum brightness projection of multilayers based on *z*-axis from confocal microscopy. Red: albumin; Blue: nucleus. Zoomed images: monolayer confocal images of regions #1-#6 in **a** and #1 in **b**. Scale bars: 30 μm in initial images and 5 μm in zoomed images. The numbers of albumosomes per view were measured (**d**). Diameters of albumosomes in 10-month-old WT mice liver sections were measured (**e**) (*n* = 4–6). **f**–**h** IF of endogenous albumin in HepG2-WT. Representative images were shown. The ratio of albumin intensity of albumosome/non-albumosome in one cell was measured (**h**) (*n* = 3). Scale bar: 5 μm. Data are analyzed by unpaired two-tailed Student’s *t* test and represented as mean ± SEM. ***p < 0.001, ****p < 0.0001

Here, we explored the intracellular localization of albumin in liver sections from mice at different ages. Surprisingly, we found that some spherical subcellular structures with smooth surface were present in the livers of 10-month-old WT mice, and the outer layer of these spheres was composed of albumin forming a shell (Fig. 2a). Since this kind of intracellular albumin has never been reported previously, we name this globular shell that is formed by albumin as “albumosome”. From the perspective of aging, the livers from 10-month-old mice contained the highest numbers of albumosomes (Fig. 2a), followed by the livers from 8-week-old mice (Fig. 2b); however, these structures were almost invisible in the livers of 3-week-old mice (Fig. 2c). This means that albumosomes accumulate with aging (Fig. 2d), which is consistent with the finding that WT livers were more resistant to fatty liver than AKO livers at an older age (Fig. 1d, e); these data suggest that intracellular albumin may regulate mitochondrial homeostasis and lipid metabolism through albumosomes. We measured the size of the albumosomes and found that their average diameter was 4 μm (Fig. 2e). ER- or Golgi-derived vesicles have been reported to be no larger than 300 nm, in diameter;^57,58^ obviously, based on both morphology and size, albumosomes are not ER- or Golgi-derived vesicles. From a wider variety of liver sections, we found it interesting that albumosomes located as clusters in the liver, and the albumosomal-rich regions were close to the edge of the liver (Supplementary Fig. S3).

In addition to the liver sections, we also observed albumosomes in HepG2 cells cultured in growth medium (Fig. 2f, g), and the morphology was consistent with that observed in the liver (Fig. 2a). In line with the liver sections, not all HepG2 cells have visible albumosomes when observed by the confocal microscope. Moreover, we calculated the proportion of albumosomes to the total amount of intracellular albumin and found that albumosomes accounted for approximately one-third of the albumin present in the cells (Fig. 2h). These findings demonstrate a novel intracellular form of albumin, namely, the albumosome.

### Pre-folding albumin undergoes phase transition to form albumosomes

**Fig. 3:**
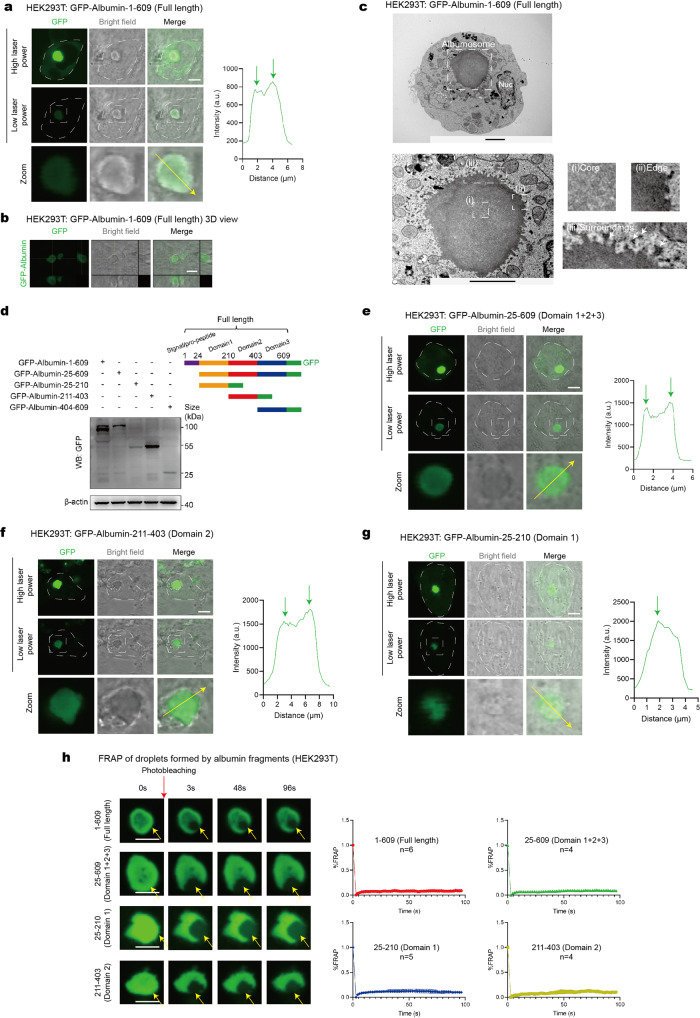
Pre-folding albumin undergoes phase transition to form albumosomes. **a**, **b** GFP-Albumin-1–609 (Full length) was transfected in HEK293T. Albumosomes were observed and the intensity along the yellow arrow was measured (**a**). 3D images were shown (**b**). Representative images were shown. Scale bars: 5 μm. **c** TEM of the albumosome in HEK293T. GFP-Albumin was transfected into HEK293T cultured in a gridded glass bottom dish for 48 h and was then fixed. Combined with light microscopy and TEM, the specific location of cells with albumosomes was obtained and the ultrastructure of albumosomes was observed. (i) Representative image of the core of the albumosome. (ii) Representative image of the edge of the albumosome. (iii) Representative image of the surroundings of the albumosome. Scale bars: 2 μm. **d** Mapping of different regions of albumin. Western blot results of different fragments of albumin in HEK293T. **e**–**g** Confocal microscopy of HEK293T transfected with GFP-Albumin-25–609 (Domain 1 + 2 + 3) (**e**), GFP-Albumin-211–403 (Domain 2) (**f**), and GFP-Albumin-25–210 (Domain 1) (**g**). Representative images were shown. Scale bars: 5 μm. **h** FRAP of droplets formed by each albumin fragment. Representative images were shown. The relative recovery intensity rates were analyzed (*n* = 4–6). Scale bars: 5 μm. Data are analyzed by unpaired two-tailed Student’s *t* test and represented as mean ± SEM

To investigate the mechanism by which albumosomes form in the cytoplasm, a plasmid vector containing full-length albumin fused with green fluorescent protein (GFP) was constructed and transfected into HEK293T cells. As expected, using confocal microscopy, we observed shell-like green droplets, which also had spherical structures according to bright field images (Fig. 3a, b). Since the fluorescence intensity in the albumosomal region is much higher than that in other intracellular regions, we captured cell images at two different laser powers (Fig. 3a). The higher laser power was used to show the morphology of the whole cell, and the lower laser power was used to clearly show the details of the albumosomes (Fig. 3a). Combined with light-electron microscopy, we identified the ultrastructure of albumosomes (Fig. 3c). In details, the protein density at the core was significantly lower than that at the edge of the albumosome, which is consistent with the shell-like morphology (Fig. 3c(i, ii)). Moreover, a considerable amount of small and irregular protein aggregates surrounded the albumosome (Fig. 3c(iii)), indicating that these protein aggregates act as building blocks of the albumosome, and the albumosome stays at a dynamic state, with continuous formation, maturation, and dissociation. After the transfection of GFP-Albumin into HEK293T, albumin droplets formed and increased in size in a time-dependent manner (Supplementary Fig. S4a). Thirty-six hours after transfection, the average droplet diameter became 4 μm (Supplementary Fig. S4a), which is consistent with the sizes of the albumosomes observed in mouse livers (Fig. 2e). Thus, we propose that the regular spherical shell-like albumin droplets with a diameter of approximately 4 μm are mature albumosomes, and the formation process is called albumosomal maturation (Supplementary Fig. S4a). Those small and irregular albumin aggregates are called immature albumosomes. In a wider range of view of HEK293T transfected with GFP-Albumin, we found that about 20% cells had mature albumosomes (Supplementary Fig. S4b). Besides, some cells had a considerable amount of immature albumosomes with a smaller size inside the cytoplasm (Supplementary Fig. S4b). In the cells that had albumosomes, we found that the number of mature albumosomes in each cell ranged from one to five, and most cells had one mature albumosomes (Supplementary Fig. S4b). Besides, we also tested the albumin expression levels in GFP-Albumin-transfected HEK293T and compared it with endogenous albumin protein levels in HepG2. Since the GFP-Albumin level in HEK293T was not higher than the endogenous albumin level in HepG2, it excluded the possibility that the formation of albumosome in HEK293T might be due to transfected protein overload (Supplementary Fig. S4c).

Since serum albumin is soluble in the blood and does not form any droplets even at extremely high concentrations of 35–50 g/L,^59,60^ it is interesting how albumin undergoes droplet formation intracellularly. Thus, we generated a vector expressing only amino acids (aa) 25–609 of albumin, which lacks the signal peptide at the N terminus, and transfected this construct into HEK293T (Fig. 3d). Unexpectedly, albumosome formation was observed once again (Fig. 3e), demonstrating that albumin formed albumosomes before entering the ER. Therefore, the albumosome is composed of pre-folding albumin.

Then, we generated constructs expressing each of the three domains of albumin^61,62^ to explore the mechanisms underlying albumosomal formation. As shown, domain 1 and domain 2 were stably expressed after transfection into HEK293T, but domain 3 (aa 404–609) was not (Fig. 3d). From the confocal images, it is obvious that the albumosomes formed by domain 2 were similar to those formed by the full-length protein and had a shell-like structure (Fig. 3f). Although domain 1 could form puncta in the cytoplasm, they did not have the shell-like structures (Fig. 3g). These results indicated that the sequence in domain 2 is necessary and sufficient for the formation of albumosomes. Notably, we also investigated the disordered score of the albumin sequence, and the results showed that domain 2 had the most disordered region (Supplementary Fig. S4d), which was consistent with the fact that domain 2 could form albumosomes by itself (Fig. 3f). A fluorescence recovery after photobleaching (FRAP) assay was used to investigate the dynamics of albumosomes.^26,27^ After photobleaching, the albumosomes could recover to only approximately 10% of their initial fluorescence intensity, which means that albumosomes are gel-like structures with poor dynamics (Fig. 3h). Similarly, the albumosomal aggregates formed by albumin fragments could also not be recovered into the initial ones (Fig. 3h).

### Albumosomes interact with CPT2 in the cytoplasm

**Fig. 4:**
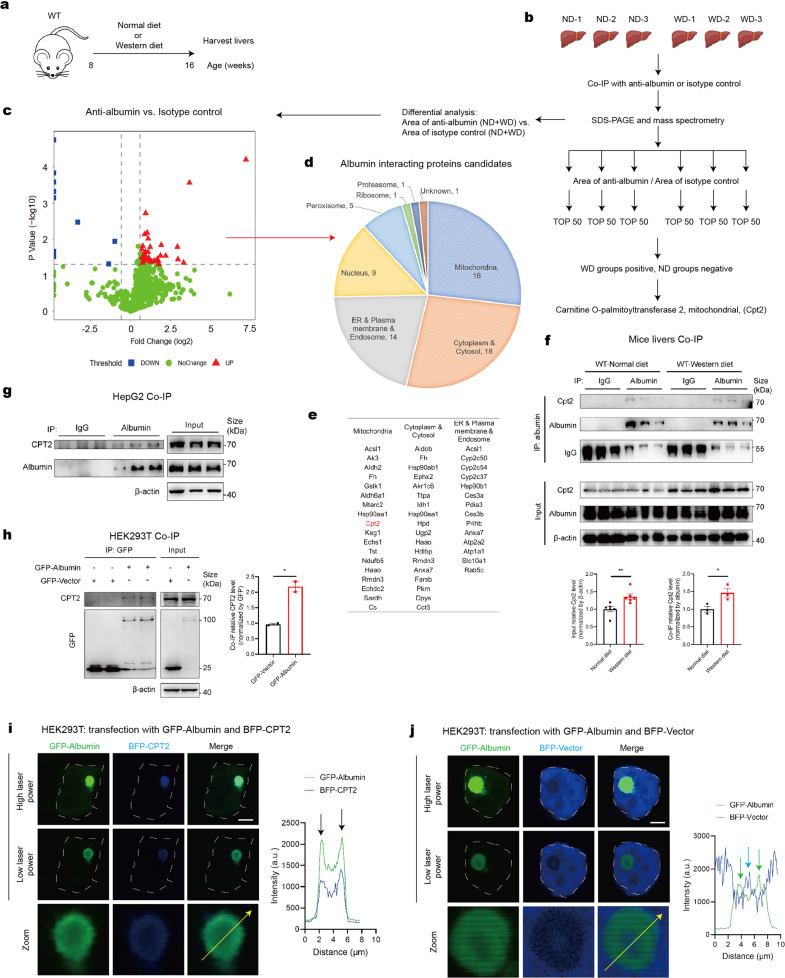
Albumosomes interact with CPT2 in the cytoplasm. **a** Experimental design model. WT mice were fed by the normal or Western diet for 8 weeks before their livers were harvested (*n* = 3). **b** Screening flow of the results of Co-IP and MS assays (*n* = 3). **c** Volcano plots of proteins in the MS result, anti-albumin vs. isotype control. The proteins that have more than 50% difference between the two groups with *p* < 0.05 were considered to be potential candidates. **d**, **e** Proteins that meet the criteria were classified by cellular localization. **f** Western blot validation of the interaction between albumin and Cpt2 in livers. Representative images were shown. Relative intensity was measured from western blot results. **g** Western blot results that show the interaction between endogenous albumin and endogenous CPT2 in HepG2. Representative images were shown (*n* = 3). **h** Western blot results that show the interaction between transfected albumin and endogenous CPT2 in HEK293T. Representative images were shown. Relative intensity was measured from western blot results (*n* = 2). **i**, **j** Confocal microscopy of HEK293T transfected with GFP-Albumin and BFP-CPT2 (**i**) or BFP-Vector (**j**). Representative images were shown. The intensities along the yellow arrows were measured. Scale bars: 5 μm. Data are analyzed by unpaired two-tailed Student’s *t* test and represented as mean ± SEM. *p < 0.05, **p < 0.01

Next, we studied the specific functions of albumosomes in the hepatic metabolic system by exploring the interactome of albumosomes in mouse liver tissues. Since a Western diet (WD) is a common model for establishing fatty liver disease,^63^ mice were fed with a normal diet (ND) or a WD for 8 weeks, and their livers were harvested (Fig. 4a). As expected, albumosomes also existed in WD mice liver sections (Supplementary Fig. S5a). Combined with co-immunoprecipitation (Co-IP) and mass spectrometry (MS) (Fig. 4b, S5b), proteins that interacted with albumin in the livers were identified and are presented as a volcano plot (Fig. 4c). Fifty proteins were identified as potential candidates (red plots) that interact with albumin in mouse livers (Fig. 4c). Then, we clustered these proteins by subcellular compartments and found that, interestingly, the most enriched compartment was the mitochondria (Fig. 4d, e). Since albumin has not been reported to be present in the mitochondria, we wondered how albumin could interact with 18 mitochondria-specific proteins (Fig. 4d, e). Therefore, it is reasonable to hypothesize that these mitochondrial proteins interact with albumosomes in the cytoplasm before they are sorted to the mitochondria.

Next, we found that among the 18 candidates, Cpt2 was the only one that met another set of screening criteria, namely, showing greater interaction with albumin in the WD group than in the ND group (Fig. 4b and Supplementary Fig. S5c). As expected, the physical interaction in mouse livers was validated by western blot (Fig. 4f). Under the stress caused by the consumption of WD, the hepatic expression of Cpt2 was upregulated (Fig. 4f), which may enhance the interaction between Cpt2 and albumin in the cytoplasm. Consistently, albumin binds to CPT2 in not only HepG2 (endogenous albumin) but also HEK293T transfected with albumin (exogenous albumin) (Fig. 4g, h), indicating that the albumin-CPT2 interaction is conserved from mice to humans. We also tested the interactions in different ages of mice livers, and found that albumin-CPT2 interaction existed in both 8-week- and 10-month-old mice livers (Supplementary Fig. S5d). Moreover, we evaluated the fatty acid oxidation and mitochondrial respiratory capacity in AKO mice fed with WD by using the GSEA analysis. Compared with AKO mice fed with ND, the liver tissues of AKO-WD exhibited significantly higher level of fatty acid β-oxidation (Supplementary Fig. S5e). Besides, AKO-WD mice showed significantly higher levels of mitochondrial respiratory capacity than WT-WD mice, indicating that AKO triggered excessive metabolic pressure on the mitochondria under the stress derived from WD (Supplementary Fig. S5f).

Furthermore, to verify the hypothesis that albumin-interacting mitochondrial proteins interact with albumosomes, we cotransfected GFP-Albumin and BFP-CPT2 into HEK293T to explore their subcellular localization. Surprisingly, we observed perfect colocalization of albumosomes and CPT2 (Fig. 4i). Upon interaction with albumosomes, BFP-CPT2 was trapped to form a spherical shell as well (Fig. 4i). On the other hand, as a negative control, BFP-Vector alone showed no colocalization with GFP-Albumin (Fig. 4j). Notably, some BFP-Vector was incorporated in the core of albumosomes (Fig. 4j), which was probably wrapped in during the process of albumosomal formation, suggesting the core of albumosomes might contain some unspecific cytoplasmic proteins and other biomolecules. To exclude the possible interference caused by the fused fluorescent protein species, mcherry-fused albumin and GFP-fused CPT2 were transfected into HEK293T, and it was shown that the albumosome also formed and trapped CPT2 in it, demonstrating that albumosomal formation is irrelevant with the fused fluorescent proteins (Supplementary Fig. S6a).

In addition to the interaction between albumosomes and CPT2 in HEK293T, we also stained for both Cpt2 and albumin in 10-month-old mouse liver sections and found that Cpt2 colocalized with albumosomes (Supplementary Fig. S6b); these results demonstrate that albumosomes trap CPT2 not only in cultured cells but also in mice livers.

### Albumosomes regulate CPT2 sorting to mitochondria to maintain mitochondrial homeostasis

**Fig. 5:**
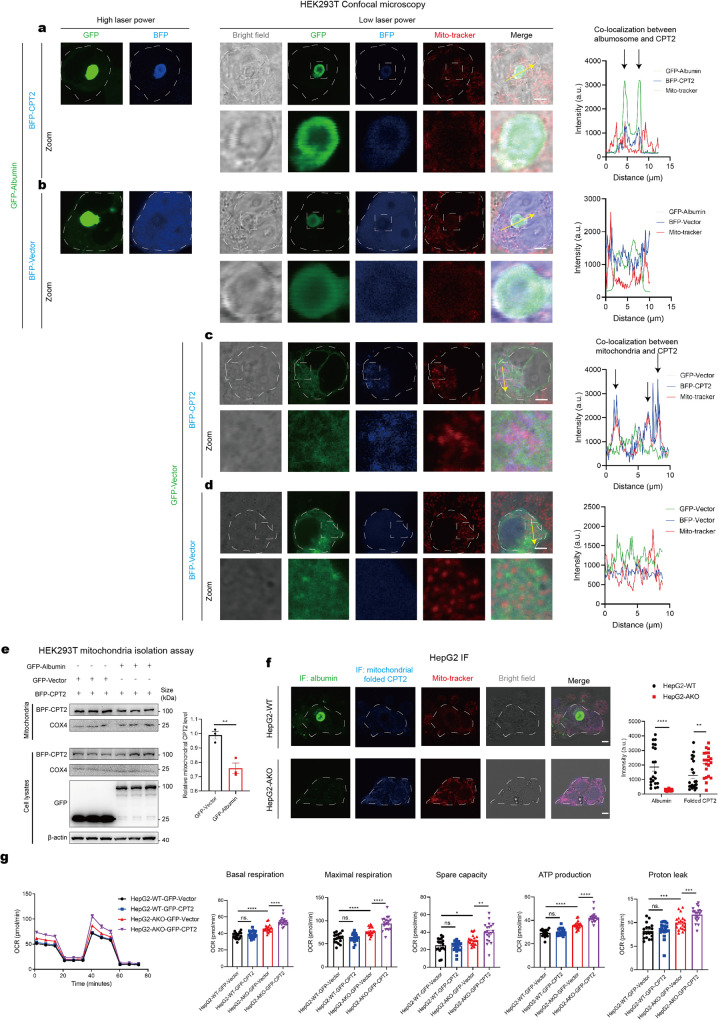
Albumosomes regulate CPT2 sorting to mitochondria to maintain mitochondrial homeostasis. **a**–**d** Confocal microscopy of HEK293T transfected with GFP-Albumin and BFP-CPT2 (**a**), GFP-Albumin and BFP-Vector (**b**), GFP-Vector and BFP-CPT2 (**c**), GFP-Vector and BFP-Vector (**d**). Mito-tracker was used for mitochondria staining. Representative images were shown. The intensities along the yellow arrows were measured. Scale bars: 5 μm. **e** HEK293T mitochondria isolation assay. Mitochondria was isolated from HEK293T transfected with GFP-Albumin and BFP-CPT2 or GFP-Vector and BFP-CPT2. Relative intensity was measured from Western blot results (*n* = 3). **f** IF of albumin and mitochondrial folded CPT2 of HepG2-WT and HepG2-AKO. Mito-tracker was used for mitochondria staining. Representative images were shown. Intensities of albumin and folded CPT2 were measured. Scale bars: 5 μm. **g** Seahorse mitochondrial respiration assay of HepG2-WT and HepG2-AKO overexpressed with GFP-Vector or GFP-CPT2, respectively. Basal respiration, maximal respiration, spare capacity, ATP production, and proton leak were analyzed. Data are analyzed by unpaired two-tailed Student’s *t* test and represented as mean ± SEM. ns. represents no significance. *p < 0.05, **p < 0.01, ***p < 0.001, ****p < 0.0001

As a mitochondrial protein that is synthesized in the cytoplasm, CPT2 is sorted to the mitochondria *via* a signal peptide (aa 1–25) at the N terminus and then folded inside the mitochondria.^64,65^ Thus, to test whether albumosome-trapped CPT2 is in a folded or pre-folding state, we generated a CPT2 construct that lacked the signal peptide (aa 26–658), meaning that it could not be sorted into the mitochondria, and was fused to FLAG (Supplementary Fig. S7a). As expected, FLAG-CPT2-26-658 also showed a physical interaction with albumin in the Co-IP assay (Supplementary Fig. S7a), demonstrating that albumosomes trap pre-folding but not folded CPT2. In addition, the FLAG-CPT2-179-658 fragment exhibited similar interactions, while FLAG-CPT2-26-209 exhibited no such interaction (Supplementary Fig. S7a); these results indicated that the binding site is located in the C terminus. Based on the interaction between pre-folding CPT2 and albumosomes, we investigated whether albumosomes affect the sorting of CPT2. By analyzing the subcellular localization relationship between CPT2 and mitochondria, we found that in the presence of albumosomes, CPT2 was trapped in the cytoplasm, and this portion of CPT2 could not enter the mitochondria freely (Fig. 5a, b). In contrast, almost all CPT2 was distributed to the mitochondria when there were no albumosomes (Fig. 5c, d). Moreover, albumosomes did not colocalize with mitochondria (Fig. 5a, b), which also indicated that albumosomes did not interact with folded CPT2.

In addition, through a mitochondria isolation assay, we found that albumosomes reduced the content of transfected BFP-CPT2 in mitochondria (Fig. 5e). Consistently, the intensity of mitochondrial CPT2 staining in the HepG2-WT was lower than that in the HepG2-AKO according to IF analysis (Fig. 5f and Supplementary Fig. S7b). These findings demonstrate that albumosomes bind and trap pre-folding CPT2 in the cytoplasm and prevent it from being sorted into the mitochondria.

Moreover, the Co-IP-MS assay in mice livers informed us that Cpt2 should not be the only protein that interacts with albumosomes (Fig. 4e), therefore, we chose another two proteins, Acsl1 and Fh, which had relatively high ranks (the first and the fourth, respectively) in the MS results, and we investigated their interactions with albumosomes. Cotransfection of BFP-ACSL1 and GFP-Albumin showed that BFP-ACSL1 was colocalized with and trapped in the albumosomes (Supplementary Fig. S8a). Besides, the physical interaction between ACSL1 and albumin was also validated by the Co-IP assay in HEK293T (Supplementary Fig. S8b). Expectedly, FH also interacted with albumin and was trapped by albumosomes (Supplementary Fig. S8c, d). It is well-accepted that ACSL1 plays a key role in both fatty acid β-oxidation and fatty acid synthesis in the liver,^66,67^ and FH is an essential enzyme catalyzing the hydration of fumarate to L-malate in the TCA cycle in mitochondria.^68^ Thus, these findings indicated that another two key proteins in metabolic pathways (fatty acid β-oxidation, fatty acid synthesis, and TCA cycle), namely, ACSL1 and FH, might be influenced by albumosomes similarly to CPT2.

To examine whether albumosomes affect mitochondrial respiration by regulating CPT2 sorting and fatty acid β-oxidation, CPT2 was transiently silenced by siRNA in HepG2-AKO (Supplementary Fig. S8e). As expected, we observed decreased mitochondrial basal respiration, maximal respiration, spare capacity, ATP production and proton leak levels after CPT2 silencing (Supplementary Fig. S8f), but these levels in the HepG2-AKO-siCPT2 cells were still higher than those of the HepG2-WT cells (Supplementary Fig. S8f). Moreover, we overexpressed CPT2 in both HepG2-WT and HepG2-AKO cells (Supplementary Fig. S8g). Using the seahorse mitochondrial respiration assay, we found that CPT2 overexpression did not have significant impact on HepG2-WT cells, but triggered significant increased levels of respiration in HepG2-AKO cells (Fig. 5g). These findings indicated that albumosomes in HepG2-WT cells control the sorting of excessive newly-synthesized pre-folding CPT2 to maintain the mitochondrial respiration, but the excessive CPT2 proteins in HepG2-AKO cells move more freely and are sorted to mitochondria directly to induce the mitochondrial respiration. To test if CPT2 is the downstream of ACSL1 or FH, we overexpressed or knocked down ACSL1, FH, or both and checked the CPT2 expression. The results showed that CPT2 did not significantly change in either loss or gain of function of ACSL1 or FH (Supplementary Fig. S8h). These data demonstrate that albumosomes maintain mitochondrial homeostasis partially by regulating CPT2 sorting and fatty acid β-oxidation. It has recently been reported by the Li group that knockout of Cpt2 influenced the fatty acid β-oxidation level in AML12 murine hepatocyte cell line,^69^ which is also consistent with the findings in this study.

### Hsp90 and Hsp70 family proteins surround albumosomes and inhibit albumosomal accumulation in an ATPase-dependent manner

**Fig. 6:**
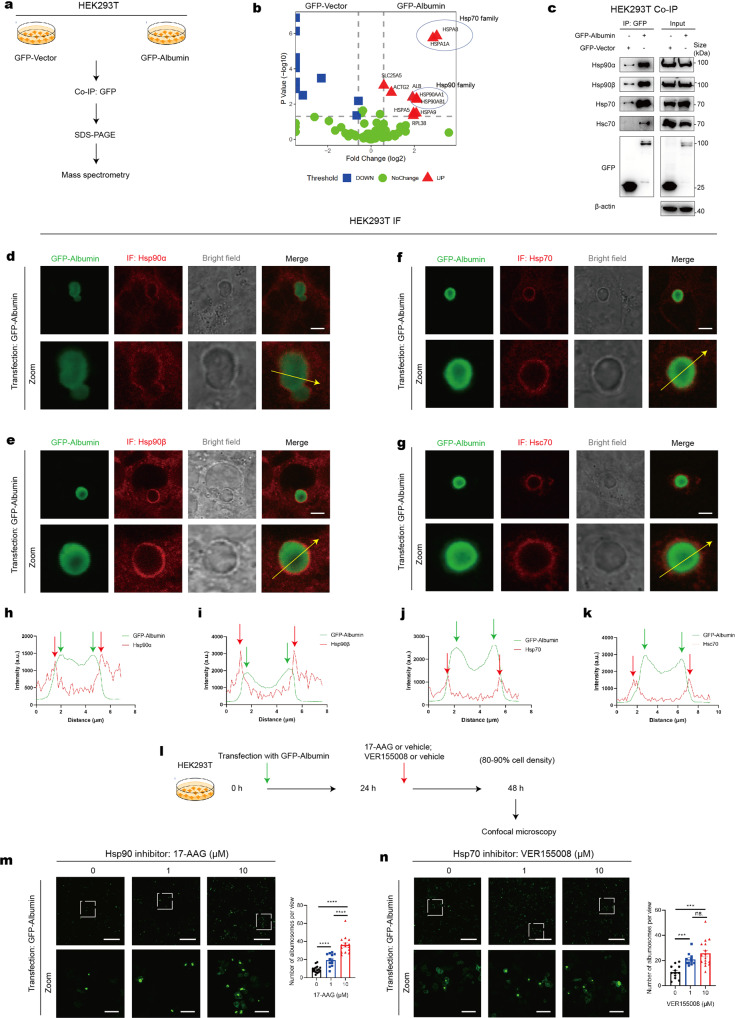
Hsp90 and Hsp70 family proteins surround albumosomes and inhibit albumosomal accumulation in an ATPase-dependent manner. **a** Experimental design model. Co-IP and MS in HEK293T transfected with GFP-Albumin or GFP-Vector (*n* = 3). **b** Volcano plots of proteins in MS, GFP-Albumin *vs*. GFP-Vector. The proteins which have more than 50% difference between the two groups with p < 0.05 were considered to be potential candidates. **c** Western blot validation of the interaction between GFP-Albumin and Hsp90 and Hsp70 family proteins. **d**–**g** IF of Hsp90α (**d**), Hsp90β (**e**), Hsp70 (**f**), and Hsc70 (**g**) in HEK293T around the albumosomes. Representative images were shown. Scale bars: 5 μm. **h**–**k** Intensity curves along the yellow arrows, (**h**) for (**d**), (**i**) for (**e**), (**j**) for (**f**), and (**k**) for (**g**). **l** Experimental design model. HEK293T transfected with GFP-Albumin was treated with Hsp90i (17-AAG) or Hsp70i (VER155008). **m**, **n** Microscopy of albumosomes under treatments of 17-AAG (**m**) and VER155008 (**n**). Representative images were shown. Number of albumosomes per view were measured. Scale bars: 300 μm in initial images and 50 μm in zoomed images. Data are analyzed by unpaired two-tailed Student’s *t* test and represented as mean ± SEM. ns. represents no significance. ***p < 0.001, ****p < 0.0001

To further explore the mechanisms underlying albumosomal formation, we used a Co-IP assay to identify the proteins that interact with albumosomes in HEK293T cells. GFP-Albumin was transfected into HEK293T cells for 48 h to form albumosomes, after which co-IP of GFP and MS were followed (Fig. 6a). Based on the MS results, we found that many heat shock proteins were significantly enriched (Fig. 6b). The interactions between albumin and Hsp90/Hsp70 were further validated by western blot (Fig. 6c). Hsp90 and Hsp70 family chaperones mainly function in the cytoplasm,^70–72^ which means they have no chance to meet albumin according to the classical understanding. Thus, we hypothesized that Hsp90 and Hsp70 family proteins interact with albumosomes in the cytoplasm.

To test this hypothesis directly, we used IF to explore their cellular localization. Surprisingly, the IF images showed that Hsp90α (Fig. 6d, h), Hsp90β (Fig. 6e, i), Hsp70 (Fig. 6f, j) and Hsc70 (Fig. 6g, k) all wrapped around the outer surface of albumosomes, forming a larger shell. Chaperones help unfolding proteins remain stable or be degraded,^70–72^ and these findings were consistent with the fact that albumosomes are composed of pre-folding albumin.

Moreover, we investigated the relationship between Hsp90-Hsp70 and the formation of albumosomes by using inhibitors of these proteins (Fig. 6l). Unexpectedly, after Hsp90 ATPase was inhibited by 17-AAG in HEK293T transfected with GFP-Albumin, the number of albumosomes increased gradually in a dose dependent manner (Fig. 6m). Similarly, the Hsp70 ATPase inhibitor VER155008 also promoted albumosomal accumulation (Fig. 6n). After Hsp90 ATPase was inhibited in HepG2-WT cells, the accumulation of endogenous albumosomes was also observed (Supplementary Fig. S9a–c). In addition, the mean diameter of the albumosomes remained at approximately 4 μm regardless of whether 17-AAG was added (Supplementary Fig. S9d), which was consistent with the albumosomes observed in mouse hepatocytes (Fig. 2e). These findings demonstrate that Hsp90 and Hsp70 family chaperones inhibit the accumulation of albumosomes in an ATPase dependent manner. Thus, we performed a Co-IP assay with GFP-Albumin after HEK293T cells were incubated with 17-AAG to determine whether the interaction between albumosomes and CPT2 could be increased (Supplementary Fig. S9e). Expectedly, more endogenous CPT2 interacted with albumin in HEK293T cells in a 17-AAG dose-dependent manner (Supplementary Fig. S9e), which indicates that the albumosomal accumulation by 17-AAG administration may become a novel therapeutic strategy.

Since the formation of albumosomes in the cytoplasm is dynamic, we were interested in the fate of both albumosomes and albumosomal clients (CPT2, ACSL1, FH, etc.). According to a ubiquitin binding assay, neither albumin nor CPT2 was conjugated with ubiquitin in HEK293T cells transfected with both GFP-Albumin and Myc-CPT2 (Supplementary Fig. S10a). This is also consistent with the confocal microscopy images showing that albumosomes had no ubiquitin colocalization (Supplementary Fig. S10b). These data indicate that ubiquitination is not the pathway by which albumosomes are degraded. Thus, we studied the relationship between albumosomes and autophagy. However, immunofluorescence staining of LC3 showed that only a very small part (approximately 13%) of the albumosomes were surrounded by LC3 puncta (Supplementary Fig. S11a, b). These results suggest that albumosomes stay in a relatively stable state, and that the degradation of albumosomes is slow and delayed. The Ge group recently reported that CCT2 acts as an aggrephagy receptor for solid aggregates.^73^ Here, we found that no accumulation of CCT1, CCT2, CCT3, or CCT6 was around the albumosomes according to immunofluorescence staining (Supplementary Fig. S12), which also suggests that albumosomes exhibit unique characteristics and functions, different from those of toxic aggregates such as polyQ-huntingtin and FUS-P525L.^73^ Since the degradation of albumosomes is slow, the albumosomal clients such as CPT2 could be retained in the cytoplasm for a considerable time before either leaving or being degraded. According to the western blot analysis of whole HEK293T cell lysates, the level of endogenous CPT2 was similar with or without the transfection of albumin (Fig. 4h and Supplementary Fig. S9e), demonstrating that CPT2 is almost not degraded after being trapped by albumosomes; thus, we name this albumosome-mediated retention process albumoretention. Based on these findings, we hypothesize that the clients might be released from some albumosomes in a sustained manner and enter the mitochondria finally, which not only protects mitochondria from acute metabolic stress but also avoids wasting these newly synthesized pre-folding mitochondrial proteins. Since it has been validated that albumosomes have at least three clients (CPT2, ACSL1, and FH), albumoretention might be universal to many other mitochondria-localized proteins, through which the related metabolic processes are regulated.

### Inhibition of Hsp90 suppresses NAFLD progression by promoting albumosomal accumulation

**Fig. 7:**
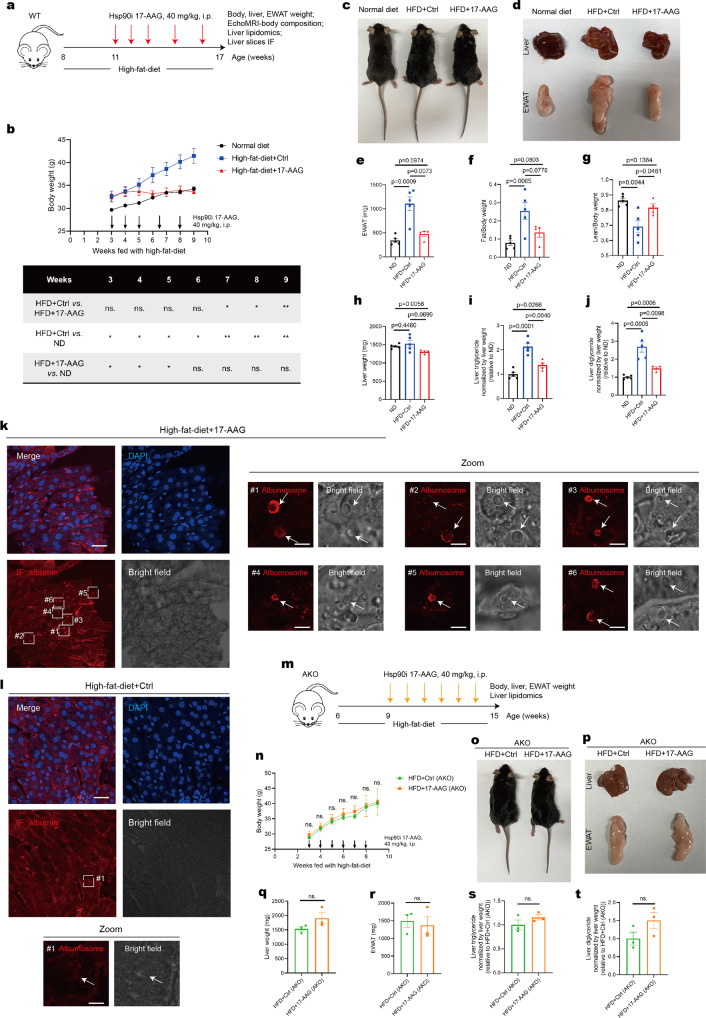
Inhibition of Hsp90 suppresses NAFLD progression by promoting albumosomal accumulation. **a** Experimental design model. WT mice fed with HFD from the age of 8 weeks were injected by 17-AAG at the 3rd, 4th, 5th, 6.5th, and 8th weeks of the HFD feeding process. The control group was injected by the solvent. Body composition was measured at the 9^th^ week, after which mice were sacrificed with tissues harvest (*n* = 4–5). **b** Body weight change of the mice in HFD + 17-AAG, HFD + Ctrl, and ND groups (*n* = 4–5). **c** Representative images of the mice from HFD + 17-AAG, HFD + Ctrl, and ND groups at the 9th week of HFD process. **d** Representative images of the livers and EWAT from HFD + 17-AAG, HFD + Ctrl, and ND groups at the 9th week of HFD process. **e**–**g** EWAT weights of HFD + 17-AAG, HFD + Ctrl, and ND groups (**e**). The ratio of fat (**f**) and lean (**g**) compared to body weight (*n* = 4–5). **h**–**j** Liver weights of HFD + 17-AAG, HFD + Ctrl, and ND groups (**h**). Relative liver triglyceride (**i**) and diglyceride (**j**) content normalized by liver weight (*n* = 4–5). **k**, **l** Liver sections of HFD + 17-AAG (**k**) and HFD + Ctrl (**l**) were used in IF. Representative images of maximum brightness projection of multilayers based on *z*-axis from confocal microscopy. Scale bars: 30 μm. Red: Albumin; Blue: nucleus. Zoomed images: monolayer confocal images of regions #1-#6 in **k** and #1 in **l**. Scale bars: 5 μm. **m** Experimental design model. AKO mice fed with HFD from the age of 6 weeks were injected by 17-AAG at the 3rd, 4th, 5th, 6th, 7th, and 8th weeks of the HFD feeding process. The control group was injected by the solvent. Mice were sacrificed with tissues harvest at the 9th week (*n* = 3–4). **n** Body weight change of the HFD + 17-AAG (AKO) and HFD + Ctrl (AKO) (*n* = 3–4). **o** Representative images of the HFD + 17-AAG (AKO) and HFD + Ctrl (AKO) at the 9th week of HFD process. **p** Representative images of the livers and EWAT in HFD + 17-AAG (AKO) and HFD + Ctrl (AKO) at the 9th week of HFD process. **q**, **r** Weights of liver (**q**) and EWAT (**r**) of the two groups, HFD + 17-AAG (AKO) and HFD + Ctrl (AKO) (*n* = 3). **s**, **t** Relative liver triglyceride (**s**) and diglyceride (**t**) content normalized by liver weight of the two groups, HFD + 17-AAG (AKO) and HFD + Ctrl (AKO) (*n* = 3). Data are analyzed by unpaired two-tailed Student’s *t* test and represented as mean ± SEM. ns. represents no significance. *p < 0.05, **p < 0.01

Since it has been verified that albumosomes trap several client proteins, through which the mitochondrial homeostasis is influenced, and Hsp90 inhibitor 17-AAG triggers albumosomal accumulation, we tried to determine whether Hsp90 inhibition could suppress NAFLD progression in a mouse model. Briefly, a HFD was fed to WT mice from the ages of 8 weeks to 17 weeks, and beginning in the third week of HFD feeding process, 17-AAG or control was injected into the mice (Fig. 7a). During this feeding process, we measured the body weights of the mice in the three groups, and found that just after the second injection, the mice in the HFD + 17-AAG group lost weight compared those in the HFD + Ctrl group, and the weights in the HFD + 17-AAG group were even similar to those in the ND group (Fig. 7b). After nine weeks of HFD feeding, the body composition of the mice was measured by Echo Magnetic Resonance Imaging (EchoMRI), and then the livers, EWAT, and serum were harvested (Fig. 7a). Consistent with the change in body weight, the weights of the EWAT in the HFD + 17-AAG group were significantly lower than those in the HFD + Ctrl group, and similar to those in the ND group (Fig. 7c–f). Reasonably, the lean contents in the HFD + 17-AAG group were significantly higher than those in the HFD + Ctrl group (Fig. 7g). Moreover, the liver weights of HFD + 17-AAG group were slightly decreased compared with those of the HFD + Ctrl group (Fig. 7h). According to the liver lipidomics analysis, we found that after 17-AAG administration, the contents of both TG and DG in the livers of the HFD + 17-AAG group were significantly lower than those in the HFD-Ctrl group (Fig. 7i, j, S13a-S13b), which was consistent with the change in fat tissue weights (Fig. 7e, f). Moreover, the liver tissues from the three groups were subjected to RNA-seq for gene expression analysis. Using the GSEA analysis, we found that HFD triggered the fatty acid β-oxidation in livers (Supplementary Fig. S13c), which probably results from excessive fat deposition in the hepatocytes. Besides, compared to ND livers, response to unfolded protein was stimulated in HFD livers (Supplementary Fig. S13d), which indicated that HFD trigger excessive production and retention of pre-folding or unfolded proteins, leading to severe unfolded protein stress to the hepatocytes. However, livers in the HFD + 17-AAG group showed significantly lower level of fatty acid β-oxidation than that in the HFD + Ctrl group (Supplementary Fig. S13e), which indicated that Hsp90 inhibition suppresses the metabolic stress in livers derived from the HFD. Besides, 17-AAG significantly decreased the genes transcription related with mitochondrial respiratory chain (Supplementary Fig. S13f, g), which suggested that Hsp90 inhibition relieved the mitochondrial stress and maintained the mitochondrial homeostasis. We also investigated the ultrastructure of mitochondria in both groups, and it showed that the ratio of hyperfused and irregular mitochondria in HFD + 17-AAG was significantly lower than that in HFD + Ctrl (Supplementary Fig. S13h), which verified the mitochondrial homeostasis in HFD + 17-AAG hepatocytes. In addition, the liver function tests were performed with serum samples, and the results showed that 17-AAG decreased the levels of alanine amino transferase (ALT) (Supplementary Fig. S13i), meanwhile other parameters were not significantly different (Supplementary Fig. S13j–n), suggesting that 17-AAG could protect livers from HFD damage without causing additional injury. These data demonstrate that 17-AAG suppressed NAFLD and obesity in the HFD mouse model.

IF staining in liver sections revealed, as expected, that more albumosomes were observed in the livers of the HFD + 17-AAG group than in the livers of the HFD + Ctrl group (Fig. 7k, l). Besides, the albumin level showed no difference in the liver tissues between HFD + Ctrl and HFD + 17-AAG (Supplementary Fig. S13o). Thus, we tried to validate whether the regulatory effect of 17-AAG on the liver lipid metabolism is mediated by albumosomal accumulation in hepatocytes. Therefore, AKO mice were also fed a HFD and injected with 17-AAG (Fig. 7m). As expected, the AKO mice showed no significant differences in body, liver, or EWAT weight regardless of whether 17-AAG or the control was injected (Fig. 7n–r). In addition, according to the lipidomics analysis of the two groups of livers, neither TG nor DG showed significant difference between the HFD + 17-AAG group and the HFD + Ctrl group in AKO mice (Fig. 7s, t). These data demonstrate that 17-AAG suppresses the progression of NAFLD and obesity by promoting albumosomal accumulation, which protects the hepatocyte mitochondria from HFD nutrient stresses.

**Fig. 8:**
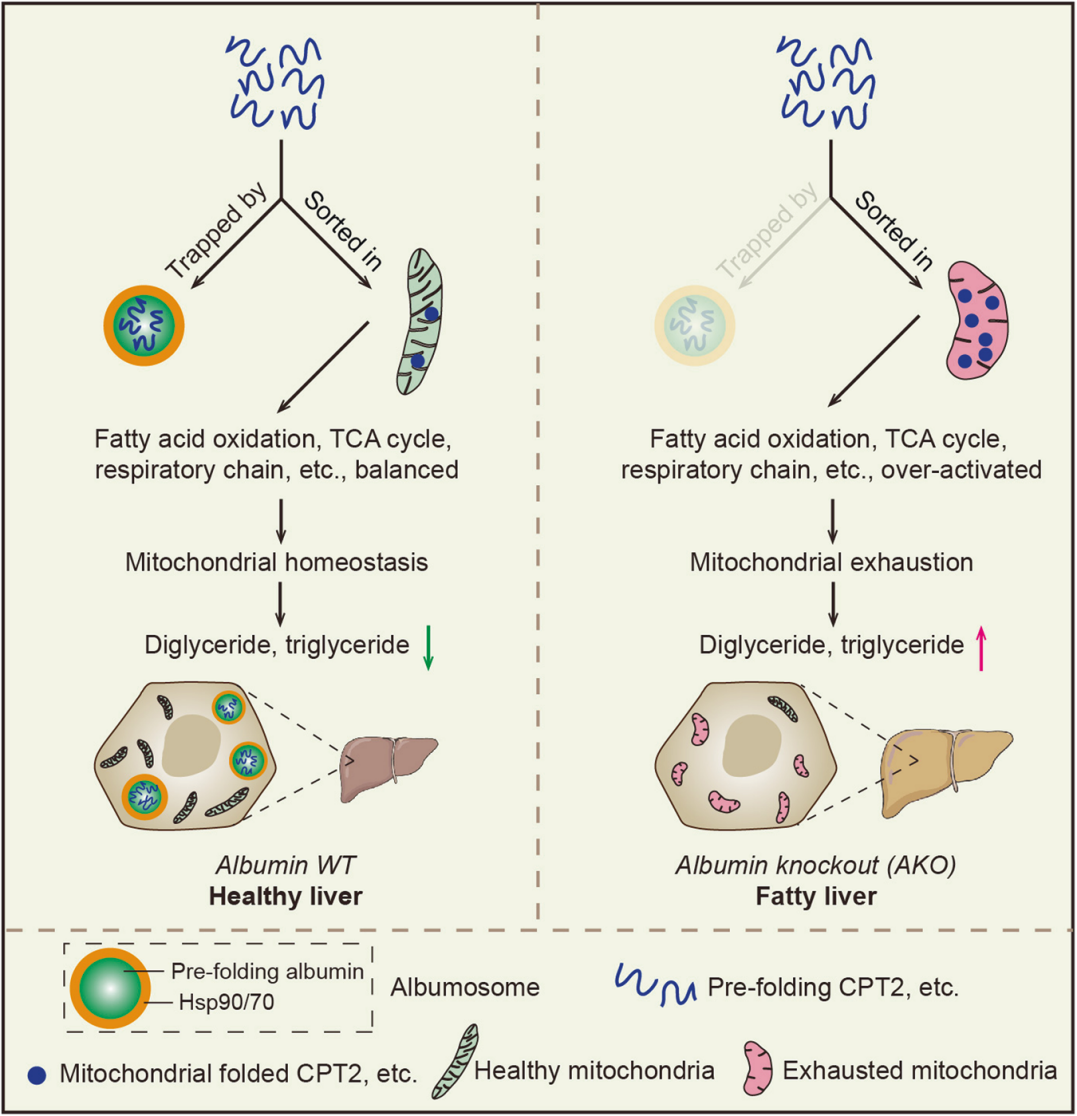
Graphic summary of this study. Stresses from aging or HFD stimulate the expression of mitochondrial proteins related with fatty acid β-oxidation and synthesis, TCA cycle, and respiratory chain, etc. In WT livers, excessive pre-folding CPT2 could be trapped by albumosomes in the cytoplasm, relieving the stress of mitochondria and maintaining lipid metabolic homeostasis, which form a virtuous cycle and inhibit fat deposition. However, in AKO livers, such stress-induced excessive pre-folding mitochondrial proteins enter mitochondria more freely lacking the capture by albumosomes, and stimulate mitochondrial overloaded respiration. Continuously, prolonged stresses aggravate mitochondrial exhaustion and result in lipid metabolic disorders, which lead to a vicious cycle and trigger fat deposition

Based on all of the findings in this study, some functions of the newly discovered albumosomes are shown in the graphic summary (Fig. 8).

## Discussion

In this study, we discovered a novel membrane-less shell-like organelle in hepatocytes that is formed *via* phase transition of the intracellular pre-folding albumin, and we named this structure the “albumosome”. Morphologically, the average diameter of mature albumosomes is 4 μm. Functionally, pre-folding CPT2 is trapped in the cytoplasm by albumosomes through physical interaction so that CPT2 cannot freely enter mitochondria. In vivo studies showed that consumption of a Western diet upregulated Cpt2 in mouse hepatocytes (Fig. 4f), however, continuous excessive mitochondrial Cpt2 leads to hyperactivation of fatty acid β-oxidation, resulting in mitochondrial damage.^74–76^ Albumosomes reduce mitochondrial exhaustion and dysfunction by controlling the entry of excess CPT2 into mitochondria, through which maintaining the mitochondrial homeostasis. Physiologically, albumosomes are surrounded by Hsp90 and Hsp70 family proteins, besides, the inhibitions of which trigger the albumosomal accumulation in the cytoplasm, and further trap more client proteins such as CPT2 staying in the cytoplasm. Thus, based on these mechanisms, Hsp90 inhibitor 17-AAG exhibits efficacy in treating the NAFLD and obesity in mice. Recently, it has been reported that inhibition of Hsp90 suppresses lipid synthesis *via* directly promoting the degradation of sterol regulatory element-binding protein 1 (SREBP1) and SREBP2.^77–79^ Here, our study proposes an independent mechanism by which the Hsp90 inhibitors function in the treatment of NAFLD and obesity.

Since the albumin-CPT2 interaction also occurs in the livers of 16-week-old mice (Fig. 4f), which have fewer albumosomes than the livers of 10-month-old mice, it is reasonable that immature albumosomes (small albumin aggregates, Fig. 3c(iii)) and even soluble pre-folding albumin itself interact with their clients such as CPT2 before the formation of mature albumosomes. However, under this circumstance, interaction with free pre-folding albumin is less able to trap client proteins and inhibit their sorting than interaction with albumosomes. When pre-folding albumin forms mature albumosomes in the livers of older mice or the livers younger mice treated with 17-AAG, because of the solid-like characteristics of albumosomes, it is difficult for the binding clients to freely dissociate form albumosomes, which allows albumosomes to play roles in mitochondrial sorting and homeostasis.

Chaperones play various roles in the process of protein phase separation.^80–83^ The Cleveland group recently reported that disease-causing TDP-43 mutations form spherical shells in the nuclei and Hsp70 proteins locate in the core of the TDP-43 anisosomal shells to maintain the structures.^28^ The Liu group also reported that Hsp70 chaperones TDP-43 in the liquid-like phase and prevents it from toxic aggregation.^80^ It has been well-established that cytotoxic aggregates such as TDP-43, SOD1, Aβ, Tau, α-synuclein, and huntingtin in the neurodegenerative diseases undergo either ubiquitin-mediated or autophagic degradation.^73,84^ However, unlike these protein aggregates, albumosomes in the cytoplasm have no ubiquitination or autophagic degradation (Supplementary Figs. S10–S12), which suggest that albumosomes are not misfolded protein aggregates and are low-toxic to the cells. Since Hsc70 is an interacting protein of albumosomes, we did not exclude the possibility that albumosomes could be degraded via microautophagy and chaperone-mediated autophagy,^85,86^ which needs to be investigated in the future. Moreover, in the present study, it has been demonstrated that albumosomes exhibit positive functions that are protective to other organelles and the cells. It is reasonable to hypothesize that with the physiological aging process, the Hsp90/70 ATPase functions become weaker and lead to albumosomal accumulation in the liver, providing additional protection for other organelles such as mitochondria by resisting various stresses. This means that albumosomal accumulation is an active biological process for hepatic metabolic homeostasis in the aged individuals.

From the perspective of albumin evolution, it is reasonable for albumin to undergo phase transition. Folded albumin has 17 pairs of disulfide bonds that are formed in the ER,^61,62^ which has an astronomical number of possible disulfide bond pairs. The folding and packaging of albumin require elaborate regulation; otherwise, the excessive accumulation of unfolded albumin may occur, resulting in substantial ER stress. Therefore, it is economic and efficient for albumin to evolve some unique sequence to allow phase transition when it cannot freely and completely enter the ER under specific stressful conditions. Since albumin is continuously expressed in the hepatocyte at an extremely high level, the phase transition process protects both albumin itself and the hepatocyte from potential huge ER stress, which is similar with the classical function of protein phase transition.^43,44^ This also explains the fact that serum albumin cannot and does not need to phase transit even at a concentration of 35–50 g/L in the blood, but intracellular pre-folding albumin forms condensates (albumosomes). Albumosomes might serve as a pool of pre-folding albumin, and Hsp90 and Hsp70 family proteins facilitate the departure of albumosomes. After leaving albumosomes, pre-folding albumin may enter the ER for folding and then the Golgi-apparatus for secretion. However, under some conditions, pre-folding albumin could also be degraded directly. We suggest that both ways may occur, depending on the state of the cells. Nevertheless, future studies will unravel the mystery.

It has recently been reported that aggresome assembly is regulated by microtubules, HDAC6, dynein, and some centrosomal proteins in cells.^87–90^ Thus, we tend to believe that the formation of albumosomes also depends on some cellular partners, since unfolded albumin could not form spherical shell-like albumosomes in a test tube. We tried to incubate human serum albumin with dithiothreitol (DTT) to break up its disulfide bonds, although a flocculent precipitate was observed, droplet- or shell-like albumosomes could not be formed (data not shown). These results indicated that mature albumosome formation is a complicated process that is regulated by some unknown cellular proteins, which are worth identifying in the future. Another future study should be the structure of pre-folding albumin in albumosomes by TEM^91^ and cryo-electron microscopy (cryo-EM)^92,93^ in vivo, which aims to explore whether albumin is completely unfolded or present in some intermediate folding states. In addition to the endogenous albumosome, our lab has recently found that exogenous young and undamaged recombinant albumin has some remarkable novel functions in improving healthspan and lifespan, and alleviating type 2 diabetes,^94,95^ which together will open new research directions with this ancient protein.

This study reveals that albumosomes bind to and trap CPT2, ACSL1, and FH in the cytoplasm, through which play roles in the hepatic metabolic system. In addition to these three proteins, we suppose that albumosomes could act as a platform by which many other newly-synthesized pre-folding proteins are retained in the cytoplasm, and during this process some biophysical and biochemical reactions might occur. As a novel membrane-less structure in hepatocytes, albumosome needs to be studied in greater depth in the future, and could probably become a new target for the treatment of many metabolic and aging-related diseases.

## Materials and methods

### Mice models

All animal experiments were approved by the Institutional Animal Care and Use Committee (IACUC) at Tsinghua University (Beijing, China) with the Animal Protocol (AP) numbers: 18-LYZ2 and 20-LYZ2.

WT (C57BL/6) were bought from Charles River; *Alb*−/− (C57BL/6J-Albem8Mvw/MvwJ, AKO) were bought from the Jackson Laboratory.^96^ AKO litters were bred as homozygotes (*Alb*−/−, male) × heterozygotes (*Alb*+/−, female) as suggested by the Jackson Laboratory. All mice were housed in a specific-pathogen-free (SPF) facility with 12 h:12 h light:dark cycles and free to get food and water. Mice were housed at room temperature (20–24 °C). Normal diet (XIETONG BIO., SWC9102), western diet (Research diet, D12079B), and high-fat-diet (Research diet, D12492) were used to feed mice. For intraperitoneal injection in mice, 17-AAG (Selleck, S1141) was dissolved by using PEG300, tween-80, and water, at the ratio of 17-AAG (pre-dissolved in DMSO):PEG300:tween-80:water=1:8:1:10 (v/v). For body composition assay, EchoMRI^TM^-100H body composition analyzer for live small animals was used to measure the masses of fat, lean in live mice at 9 am. Mice serum biochemistry was tested by automatic biochemical analyzer (ZECEN, CLS880).

### Lipidomics assay

Fifty milligrams of mouse liver were homogenized in 500 μL media (dichloromethane: methanol = 2: 1, v/v) in a 2 mL EP tube on ice. 125 μL double distilled water was added into the tube for stratification. Then the samples were vortexed for 10 s and let stand for 10 s, and repeat this step for 3 times to extract the lipids as much as possible. After that, centrifugate the tubes at 3000 rpm for 15 min. Then the bottom layer liquid of each tube was harvested with the same volume and dried by nitrogen gas flow on ice. Reverse phase chromatography was selected for LC separation using Cortecs C18 column (2.1 × 100 mm, 2.7 μm, Waters). Mobile phase A was made by mixing 400 mL HPLC-grade water containing 0.77 g of ammonium acetate with 600 mL HPLC-grade acetonitrile. Mobile phase B contained 10% ACN and 90% IPA (v/v). Gradient elution was performed at a flow rate of 0.25 mL/min, and the column oven temperature was set to 40 °C. The gradient was as follows: 0 min, 30% B; 3.0 min, 30% B; 4.5 min, 33% B; 7.0 min, 45% B; 8.0 min, 52% B; 11.0 min, 58% B; 14.0 min, 66% B; 17.0 min, 70% B; 21.0 min, 75% B; 23.0 min, 98% B; 30.0 min, 98% B; 30.5 min, 30% B; 35.0 min, 30% B.

Lipid analysis was performed on Q Exactive HF orbitrap mass spectrometer (Thermo Fisher, USA) coupled with UHPLC system Ultimate 3000 (Thermo Fisher, USA). The detailed mass spectrometer parameters are as follows: spray voltage, 3.2 kV for positive and 2.8 kV for negative; capillary temperature, 320 °C; sheath gas flowrate (arb), 35; aux gas flow rate (arb), 10; mass range (m/z), 240–2000 for positive and 200–2000 for negative. Full MS resolution, 60000; MS/MS resolution, 15000; top N, 10; NCE, 15/30/45; duty cycle, 1.2 s. Lipids were identified and quantified using Lipidsearch 4.2 (Thermo Fisher, USA). Heatmaps were generated by R studio.

### Metabolomics assay

HepG2-WT and HepG2-AKO with the same cell number cultured in 10 cm dishes were added with 2 mL 80% (v/v) methanol (pre-chilled to −80 °C) and incubated at −80 °C overnight. Then, scrape the dishes with cell scraper and transfer the cell lysate to new tubes. After that, centrifugate the tubes at 14,000 × *g* for 20 min at 4 °C, and transfer the metabolite-containing supernatant to new tubes. Then, the tubes were dried by nitrogen gas flow on ice. Next, the metabolites were used in targeted metabolomic analysis.

Targeted metabolomic experiment was analyzed by TSQ Quantiva (Thermo, CA). C18 based reverse phase chromatography was utilized with 10 mM tributylamine, 15 mM acetate in water and 100% methanol as mobile phase A and B respectively. This analysis mainly focused on TCA cycle, glycolysis pathway, pentose phosphate pathway, amino acids and purine metabolism. A 25-min gradient from 5% to 90% mobile B was used. Positive-negative ion switching mode was performed for data acquisition. The resolution for Q1 and Q3 are both 0.7FWHM. The source voltage is 3500 v for positive and 2500 v for negative ion mode. The source parameters are as follows: spray voltage: 3000 v; capillary temperature: 320 °C; heater temperature: 300 °C; sheath gas flow rate: 35; auxiliary gas flow rate: 10. Metabolite identification was based on Tracefinder search with home-built database containing about 300 compounds.

### Liver RNA-Seq

The liver samples were placed in the mortar with liquid nitrogen, and then were fully ground under liquid nitrogen to powder. The sample powder was transferred to a 2 mL EP tube containing TRIzol lysis solution (50 mg liver samples per mL lysis solution). Then shake them vigorously, mix well and let stand at room temperature for 10 min. After that, the samples were centrifugated at 10,000 rpm for 5 min at 4 °C. Then, the supernatants were aspirated into a new 2 mL EP tube, and added 200 μL choloroform/isoamyl alcohol per mL of lysate and inverted to be well-mixed. After that, the samples were centrifugated at 10,000 rpm for 10 min at 4 °C. Then, the supernatants were aspirated into a new 1.5 mL tube, and added with same volume isopropanol to mix well. Precipitation at −20 °C for 1 h. After that, the samples were centrifugated at 13,600 rpm for 20 min at 4 °C. Discard the supernatants, add 1 mL 75% ethanol, and wash the precipitate with a pipette. After that, the samples were centrifugated at 10,000 rpm for 3 min at 4 °C, discarded the residual liquid, and air dried for 3–5 min. Then, the precipitates were dissolved with 100 μL RNase-free water. RNA-sequencing was analyzed by the BGI company.^97^ TPM of genes were used to perform gene set enrichment and GSEA. Heatmaps were generated by R studio.

### Oil red O staining

Mice livers were fixed by 4% paraformaldehyde, embedded in OCT at −80 °C for 12 h, and sliced into 15 μm thick sections (Leica, CM1950). Staining was performed according to the manufacturer’s protocol (Beyotime, C0158S). In brief, the slices were incubated by washing buffer for 20 s, and stained by reagent for 20 min. After that, discard the reagent and washed by washing buffer for 30 s and then PBS for 20 s. Finally, images of the slices were taken by microscope.

### Cell lines

HepG2 and HEK293T were bought from ATCC and cultured with DMEM, 10% fetal bovine serum (FBS) and 1% Penicillin-Streptomycin (PS) in the cell culture incubator at 37 °C and 5% CO_2_. *ALB* in HepG2 was knocked out by Haixing Biosciences using the CRISPR/Cas9 system. The sequences of gRNAs were gRNA-A1: ACCTCTGGTCTCACCAATCGGGG, and gRNA-A2: CTCCATGGCAGCATTCCGTGTGG according to http://crispor.tefor.net.

### Plasmids and transfection

Plasmids pLV-C-GFPSpark-Albumin (HG10968-ACGLN) and pLV-C-GFPSpark-CPT2 (HG18044-ACGLN) were bought from SinoBiological. Plasmids pc3.1-EBFP-CPT2, pc3.1-EBFP-ACSL1, pc3.1-EBFP-FH, pc3.1–3XFlag-CPT2-26-658, pc3.1-3XFlag-CPT2-26-208, pc3.1-3XFlag-CPT2-179-658, pLV-C-GFPSpark-Albumin-25-609, pLV-C-GFPSpark-Albumin-25-210, pLV-C-GFPSpark-Albumin-211-403, pLV-C-GFPSpark-Albumin-404–609, pc3.1-mcherry-Albumin, and pCMV-MCS-Myc-CPT2 were synthesized and ligated into vectors by RuiBiotech. The sequences of siCPT2: #1: 5’−3’, sense CCAGGCUGCCUAUUCCCAATT, antisense UUGGGAAUAGGCAGCCUGGTT; #2: 5’-3’, sense GCACAGUGCUGGUGAGCUUTT, antisense AAGCUCACCAGCACUGUGCTT; siACSL1: 5’-3’, sense CCUGAAGAUCUUGCAGUAATT, antisense UUACUGCAAGAUCUUCAGGTT; siFH: 5’-3’, sense GGUGCCAAAUGAUAAGUAUTT, antisense AUACUUAUCAUUUGGCACCTT. For transfection, plasmids and Polyplus jetPRIME reagent (101000046) were mixed at the ratio of 1 μg:2 μL in jetPRIME buffer. For siRNA, it was diluted to 20 μM, and mixed with the reagent at the ratio of 5:4 (v/v) in the buffer. Then, incubate for 12 min at room temperature. After that, the mixture was added to HEK293T and incubated for 12 h in the cell culture incubator. Finally, the medium was changed to fresh growth medium.

For lentivirus package, plasmids (target plasmid: pSPAX2: pVSVG = 4:3:2) were mixed with Polyplus jetPRIME reagent (101000046) at the ratio of 1 μg:2 μL in jetPRIME buffer. Then, incubate for 12 min at room temperature. After that, the mixture was added to HEK293T and incubated for 12 h in the cell culture incubator. Then, change to fresh culture medium and incubate for another 60 h. After that, the supernatants from the dishes were collected and centrifugated at 1100 × *g* for 2 min. Then, the supernatants were filtered using 0.45 μm membrane to exclude the cell fragments of HEK293T. After that, the lentivirus-containing medium from the last step was directly added into the target cell culture plates (HepG2-WT and HepG2-AKO) and the same volume of fresh culture medium was also added, then incubated in the cell culture incubator for 24 h. After that, change to fresh culture medium and the cells were cultured for another 48 h, and gradually, some cells showed green fluorescence. Finally, the cells were subjected to flow cytometry to sort GFP positive cells, which stably overexpressed the targeted genes.

### Co-immunoprecipitation and mass spectrometry

Mice livers were homogenized in RIPA buffer (Beyotime, P0013B, (main components: 50 mM Tris (pH 7.4), 150 mM NaCl, 1% Triton X-100, 1% sodium deoxycholate, 0.1% SDS)), with 1 mM PMSF (Beyotime, ST505), and centrifugated at 14,000 × *g* for 10 min. HepG2 was homogenized in lysis buffer (Beyotime, P0013, (main components: 20 mM Tris (pH7.5), 150 mM NaCl, 1% Triton X-100)), with 1 mM PMSF (Beyotime, ST505), and centrifugated at 14,000 × *g* for 10 min. Supernatants of mice livers and HepG2 were incubated by protein-G agarose beads (Roche, 11243233001) on a rotator for 2 h at 4 °C. Then they were centrifugated at 1000 × *g* for 1 min. The supernatants were transferred a new 1.5 mL EP tube and incubated with anti-albumin IgG (Abcam, ab207327) or isotype control IgG on a rotator for 12 h at 4 °C, followed by incubated by protein-G agarose on a rotator for 4 h at 4 °C. After that, centrifugate the tube and discard the supernatants, then add 1 mL PBS into the tube and vortex the tube for 10 s to wash the beads. The washing process was repeated for 4 times and then the beads were heated at 95 °C for 5 min in SDS loading buffer. After heating in SDS loading buffer, the agaroses beads were centrifugated at 1000 × *g* for 1 min and the supernatants were used in SDS-PAGE.

HEK293T transfected by indicated vectors were homogenized with lysis buffer (Beyotime, P0013, (main components: 20 mM Tris (pH7.5), 150 mM NaCl, 1% Triton X-100)) with PMSF (Beyotime, ST505) and centrifugated at 14,000 × *g* for 10 min. Supernatants were incubated with anti-GFP nanobody agarose beads (KT Health, KTSM1301) or anti-Myc nanobody agarose beads (KT Health, KTSM1306) in a 1.5 mL EP tube on a rotator for 2 h at 4 °C. After that, centrifugate the tube and discard the supernatants, then add 1 mL PBS into the tube and vortex the tube for 10 s to wash the beads. The washing process was repeated for 4 times and then the beads were heated at 95 °C for 5 min in SDS loading buffer. After heating in SDS loading buffer, the agaroses beads were centrifugated at 1000 × *g* for 1 min and the supernatants were used in SDS-PAGE.

The gel bands were excised from the gel, and then reduced with 5 mM dithiotreitol and alkylated with 11 mM iodoacetamide. After that, the gel was digested by sequencing grade modified trypsin in 50 mM ammonium bicarbonate at 37 °C overnight. The peptides were extracted with 0.1%TFA in 50% acetonitrile aqueous solution for 30 min twice. Then extracts were centrifuged in a speedvac to reduce the volume. Peptides were redissolved in 0.1% TFA and analyzed by LC-MS/MS.

For LC-MS/MS analysis, the peptides were separated by a 60 min gradient elution with a Thermo-Dionex Ultimate 3000 HPLC system at a flow rate of 0.30 µL/min, which was directly interfaced with a Thermo Scientific Orbitrap Fusion mass spectrometer. The analytical column was a home-made fused silica capillary column (75 µm ID, 150 mm length; Upchurch, Oak Harbor, WA) packed with C18 resin (100 Å, 2 µm, Varian, Lexington, MA). Mobile phase A: 0.1% formic acid. Mobile phase B: 80% acetonitrile and 0.1% formic acid. The mass spectrometer was operated in the data-dependent acquisition mode using Xcalibur 2.1.2 software. There was a single full-scan mass spectrum in the orbitrap (300–1500 *m*/*z*, 120,000 resolution) followed by data-dependent MS/MS scans at NCE 30%.

The MS/MS spectra from each LC-MS/MS run were searched against the mus musculus (for mice livers) and homo sapiens (for HEK293T) sequence downloaded from UniProt protein database using an in-house Proteome Discoverer (Version PD1.4, Thermo-Fisher Scientific, USA). The search criteria were as follows: trypsin as enzyme; two missed cleavage sites were allowed; carbamidomethyl (C) was set as fixed modification; oxidation (M) and deamidation (N, Q, only for HEK293T) were set as variable modification; precursor ion mass tolerance was set at 20ppm for all MS acquired in an orbitrap mass analyzer; and the fragment ion mass tolerance was set at 0.02 Da for all MS2 spectra acquired. The peptide false discovery rate (FDR) was calculated using Percolator provided by PD. When the *q* value was smaller than 1%, the peptide spectrum match (PSM) was considered to be correct. FDR was determined based on PSMs when searched against the reverse, decoy database. Peptides only assigned to a given protein group were considered as unique. The false discovery rate (FDR) was also set at 0.01 for protein identification. Volcano plots were generated by using “Wu Kong” platform (https://www.omicsolution.com/wkomics/main/).^98^

### Immunofluorescence and confocal microscopy

For liver samples, they were fixed by 4% paraformaldehyde, embedded in OCT at −80 °C for 12 h, and sliced into 10 μm-thick sections (Leica, CM1950). For cultured cells, they were cultured in 3 cm dishes with a glass bottom, then fixed by 4% paraformaldehyde for 5 min, and then incubated by glycine/PBS (2 mg/mL) for 5 min. Then, samples were permeabilizated by 0.2% Triton X-100 for 5 min, and blocked by 10% goat serum for 15 min. After that, the samples were stained by specific antibodies (CPT2: ab110293, 1:250; 26555-1-AP, 1:200; Albumin: 16475-1-AP, 1:200; Hsp90α: ab2928, 1:200; Hsp90β: ab203085, 1:200; Hsp70: 10995-1-AP, 1:200; Hsc70: 10654-1-AP, 1:200; CCT1: 10320-1-AP, 1:200; CCT2: 24896-1-AP, 1:200; CCT3: 10571-1-AP, 1:200; CCT6: 19793-1-AP, 1:200; LC3: 14600-1-AP, 1:200; ubiquitin: 10201-2-AP, 1:200. These antibodies were bought from Proteintech and Abcam) overnight at 4 °C. Then, samples were washed by PBST (0.05% Tween-20) for 5 min and for 5 times. After that, samples were stained by secondary antibodies for 1 h at room temperature, washed by PBST for 5 min and for 5 times. Finally, stained by DAPI for 2 min at room temperature and washed for 5 min by PBST, samples were observed by confocal microscopy. Mitochondria was stained when cells were alive in incubator at 37 °C by mito-tracker (Beyotime: C1035, 1:300) for 20 min. VER155008 (S7751) was bought from Selleck. The images were acquired by Olympus FV3000.

### FRAP

HEK293T transfected with GFP-Albumin and the relative fragments were cultured on 3 cm dishes with glass flat. Aggregates were photobleached with 488 nm laser at 50% power for 1–3 s. Time-lapse images were acquired over the time after bleaching.

### Western blot

The cells or protein samples were solubilized with SDS loading buffer and separated with 12% SDS/PAGE gel. After that, the proteins in the gel were transferred onto PVDF membranes. After blocked by 10% fat-free milk for 20 min at room temperature, the membranes were incubated by antibodies (GFP: M20004, 1:1000; Cpt2: 26555-1-AP, 1:1000; albumin: ab207327, 1:1000; β-actin: TA-09, 1:1000; Flag: ab49763, 1:1000; Hsp90α: ab2928, 1:1000; Hsp90β: ab203085, 1:1000; Cox4: ab14744, 1:1000; Hsp70: 10995-1-AP, 1:1000; Hsc70: 10654-1-AP, 1:1000; Acsl1: 13989-1-AP, 1:1000; Fh: 11375-1-AP, 1:1000; ubiquitin: 14094 S, 1:1000; Myc: M20002, 1:1000; Sdha: 11998 T, 1:1000; Ndufv1: 11238-1-AP, 1:1000; Uqcrc1: 21705-1-AP, 1:1000; ATP8: 26723-1-AP, 1:1000. These antibodies were bought from Proteintech, Cell Signaling, Abcam, Abmart and ZSGB-Bio.) for 2 h at room temperature. After that, the membranes were washed by TBST (tween-80, 0.01%) for 6 min and for 3 times. Then, the membranes were incubated by secondary antibodies (anti-rabbit IgG, 7074, 1:2000; anti-mouse IgG, 7076, 1:2000. Cell Signaling). After that, the membranes were washed by TBST for 6 min and for 3 times. Finally, drop the luminal reagent (sc-2048, Santa cruz) on the membranes and expose them by the equipment (Tanon, 5200) for suitable time.

### Isolation of primary mouse hepatocytes

Mice at 8 weeks old were used to isolate primary mouse hepatocytes according to the reference.^99^ After anesthetizing, the liver is perfused by a pump using pre-warmed (37 °C) perfusion buffer (PBS without calcium or magnesium, 15 mM HEPES, and 0.5 mM EDTA) for 25 mL per liver at 5 mL/min. Then, the liver is digested using pre-warmed (37 °C) digestion buffer (HBSS with calcium and magnesium, 0.3 mg/mL collagenase type 4 (Worthington, LS004188), 15 mM HEPES) for 25 mL per liver at 5 mL/min. After that, the liver is transferred to a 10 cm plate containing DMEM/10% FBS/1% PS (4 °C) and gently ruptured with forceps in several locations of the liver to release free cells. Then, the cell suspension is filtered through a 70 μm cell strainer into a tube, and then centrifugated at 50 × *g* for 2 min. The supernatant is aspirated and then another 15 mL DMEM/F12 (4 °C) is added into the tube, followed by gently resuspending the cells and centrifugated at 50 × *g* for 2 min, and this step should be repeated for 3 times. After that, the cells are counted and 10,000 cells are seeded into a well of the seahorse plate with DMEM/F12/10% FBS/1% PS (the plate is pre-coated with rat collagen according to the manufacture’s protocol, Solarbio, C8062) and cultured in a cell incubator. Twenty-four hours after that, the culture medium is changed to new medium. After another 24 h, the cells are subjected to seahorse mitochondrial respiration assay.

### Mitochondrial respiration assay

Mitochondria respiration assay was performed according to the manufacture’s protocol (Agilent Seahorse XF Cell Mito Stress Test Kit). Oligomycin (2 μM), FCCP (1 μM), and Rotenone/antimycin A (1 μM) were used in this assay.

### Transmission electron microscopy

Cells or tissues were fixed with 2% paraformaldehyde and 2.5% glutaraldehyde, and then were washed for four times with 0.1 M PB buffer (pH 7.4). The cells were fixed with 1% osmic acid and 1.5% potassium ferrocyanide for 30 min, and then washed for four times with ultrapure water. Then the cells were stained with 1% uranyl acetate overnight and then washed for four times with ultrapure water. Then the samples were dehydrated in a gradual series of ethanol (in 50%, 70%, 80%, 90% for 2 min, and in 100% for three times). Then the samples were embedded in resin overnight at room temperature before being polymerized at 60 °C. After that, 70 nm thick ultrathin sections were cut using a diamond knife, and then picked up with Formvar-coated copper grids (100 mesh). Finally, the samples were visualized with transmission electron microscope (H-7650B) at 80 kV. For light-electron combined microscopy, cells were cultured in gridded glass bottom dishes (Cellvis, D35-14-1.5GI).

### Flow cytometry

HepG2-WT and HepG2-AKO growing in DMEM, 10% fetal bovine serum (FBS) and 1% Penicillin-Streptomycin were incubated with MitoSOX (Mei5bio, MF844-01, 2.5 μM) in 37 °C for 30 min. Then the cell samples were detached from the surface of the plates where they were growing by trypsinization, and were used in flow cytometry analysis as soon as possible. The intensity of PE channel of cells was analyzed by flow cytometry (BD, FACSAria).

### Mitochondria isolation assay

Cell mitochondria isolation kit (Beyotime, C3601) was used for this assay. Cells were harvested and resuspended in mitochondria isolation reagent. Then, the cells were homogenized by mechanical homogenizer for 7 times and 5 s for each time (avoiding to damaging the mitochondria). After that, the cells were centrifugated at 600 × *g* and 4 °C for 10 min. Then, transfer the supernatants to new tubes and centrifugate at 11,000 × *g* and 4 °C for 10 min. After that, harvest the precipitates and wash with by PBS for 3 times. At last, the precipitates were used in western blot.

### Statistical analysis

Statistical parameters and significance are reported in the figures and the figure legends. Statistical analyses were conducted using the unpaired two-tailed Student’s *t* test in Graphpad Prism 8 software. Data are represented as mean ± SEM. Significance is indicated by asterisks: *p < 0.05, **p < 0.01, ***p < 0.001, ****p < 0.0001, ns. not significant.

## Supplementary information

Supplementary materials

## Data Availability

RNA-Seq data that support the findings of this study have been deposited in NCBI GEO under the accession number GSE222922.
