# Supplementary material for: Albumosomes formed by cytoplasmic pre-folding albumin maintain mitochondrial homeostasis and inhibit nonalcoholic fatty liver disease
Source: Signal Transduct Target Ther. 2023 Jun 16;8:229. doi: 10.1038/s41392-023-01437-0 (PMC10272166; doi:10.1038/s41392-023-01437-0)

Supplementary Materials for

Albumosomes formed by cytoplasmic pre-folding albumin maintain mitochondrial homeostasis and inhibit nonalcoholic fatty liver disease

Boyuan Ma^1,2,3^, Anji Ju^1,2,3^, Shaosen Zhang^1,2,3,5^, Qi An^1,2,3^, Siran Xu^1,2,3^, Jie Liu^1,2,3,6^, Li Yu^4^, Yan Fu^1,2,3,*^, Yongzhang Luo^1,2,3,*^

Correspondence to: fuyan@mail.tsinghua.edu.cn (Y. F.); yluo@mail.tsinghua.edu.cn (Y. L.)

**This PDF file includes:**

Supplementary Figures. S1 to S13

Original and uncropped films of Westernblot


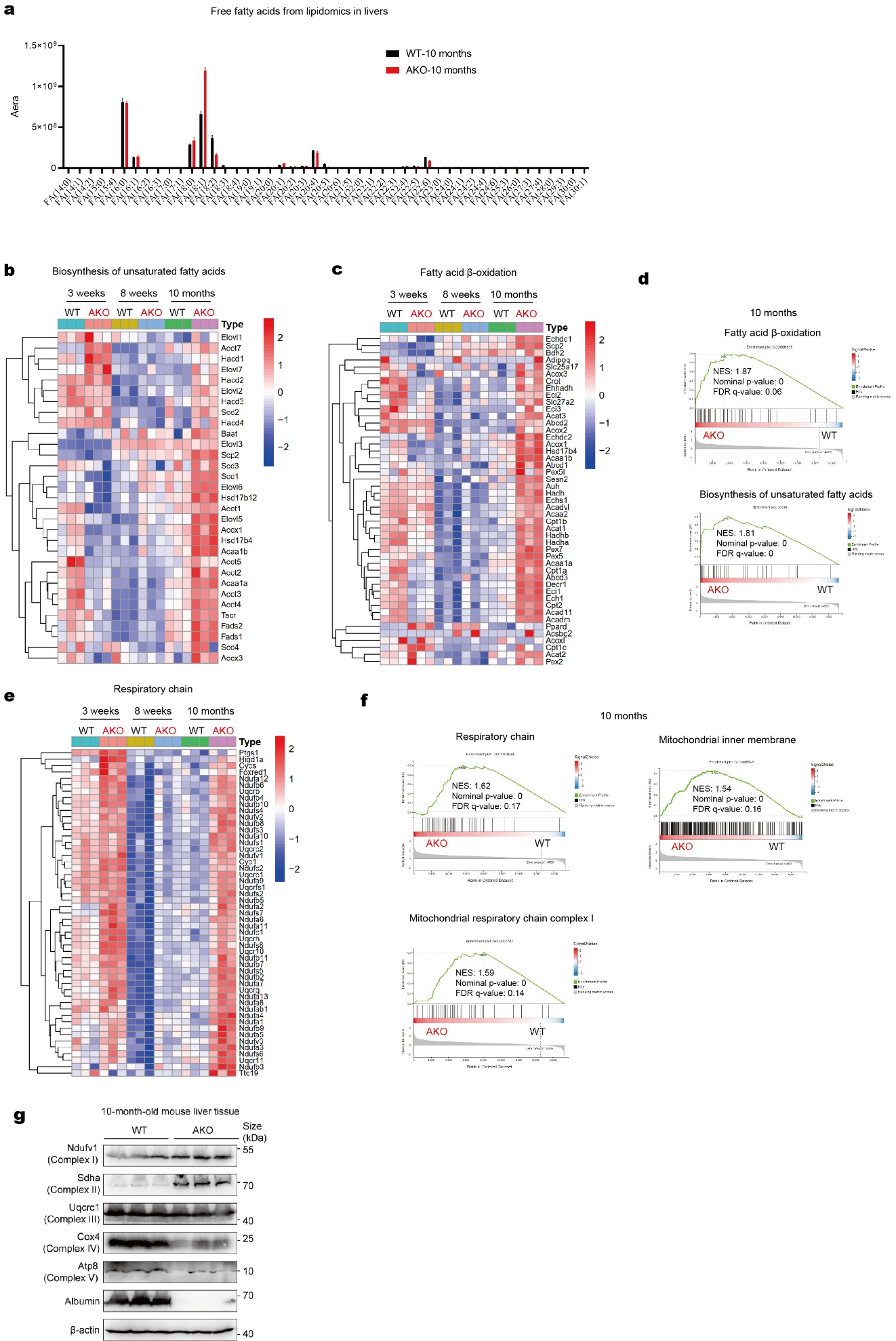


**Figure S1. Lipidomics and transcriptomics of WT and AKO mice livers.**

**a** Free fatty acids composition from lipidomics of 10-month-old WT and AKO mice (n=3). **b**-**f** Livers from WT and AKO mice of 3 weeks, 8 weeks, and 10 months of age were analyzed by RNA-Seq. Heatmap of the relative expression levels of biosynthesis of unsaturated fatty acids-related genes (**b**), and fatty acid β-oxidation-related genes (**c**). GSEA results of fatty acid β-oxidation and biosynthesis of unsaturated fatty acids of 10-month-old livers (**d**). Heatmap of the relative expression levels of respiratory chain-related genes (**e**). GSEA results of respiratory chain, mitochondrial inner membrane, and mitochondrial respiratory chain complex I of 10-month-old livers (**f**) (n=3). **g** Westernblot results of Ndufv1, Sdha, Uqcrc1, Cox4, ATP8, and albumin in 10-month-old WT and AKO livers (n=3). Data are analyzed by unpaired two-tailed student’s t test and represented as mean ± SEM.


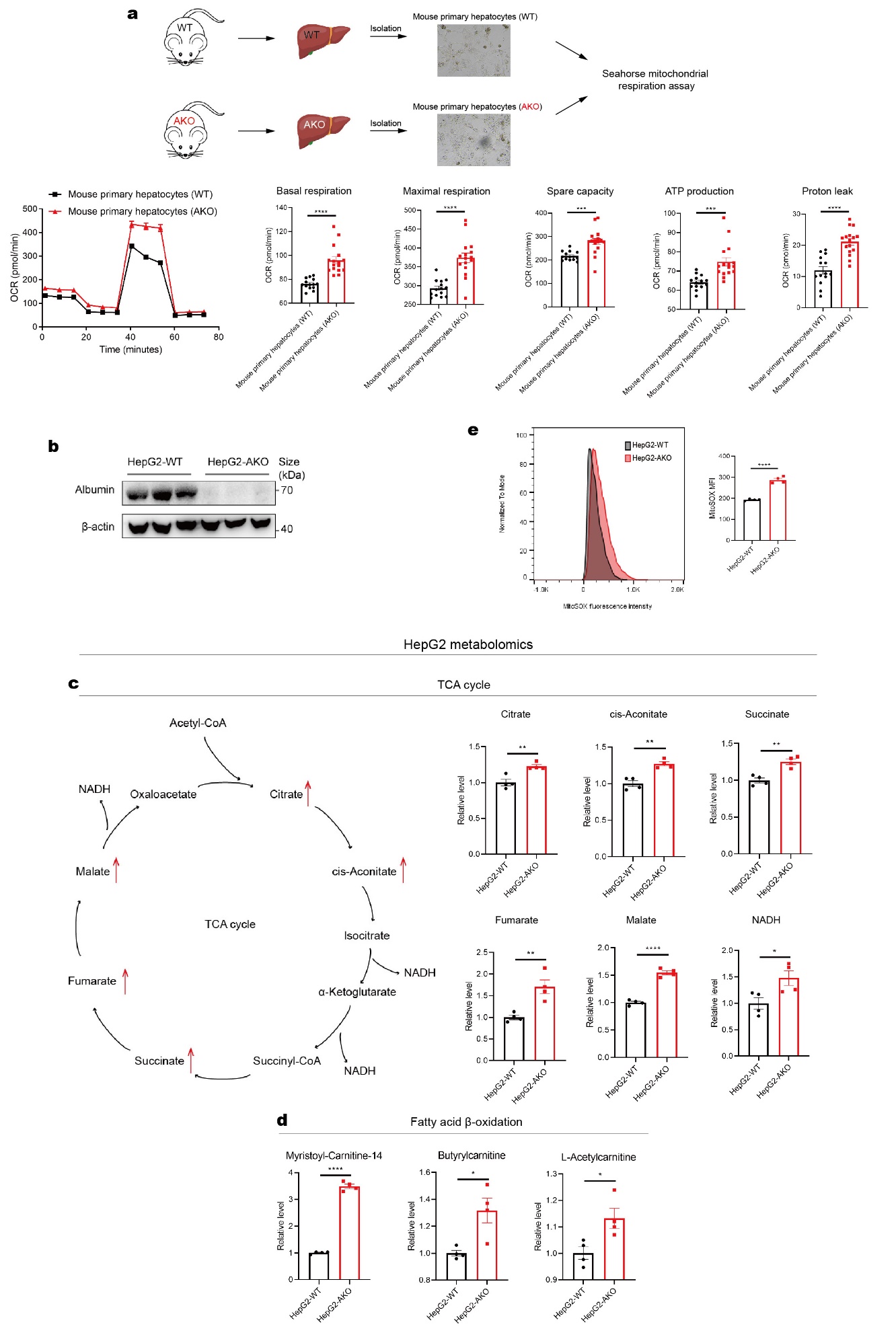


**Figure S2. Metabolic phenotypes of HepG2 and mouse primary hepatocytes with WT or AKO.**

**a** Isolation of mouse primary hepatocytes and seahorse mitochondrial respiration assay. WT and AKO mice were used to isolate mouse primary hepatocytes respectively, and basal respiration, ATP production, proton leak, maximal respiration and spare capacity were analyzed. Scale bars: 100μm. **b** Westernblot validation of albumin knockout in HepG2-AKO. **c**-**d** HepG2-WT and HepG2-AKO were used for metabolomic assay. Schema of TCA cycle related metabolites. Relative levels of these metabolites were shown, and the metabolite that with significantly higher level in HepG2-AKO than in HepG2-WT was labelled by a red arrow in the schema (**c**) (n=4). Relative levels of acylcarnitine in the metabolomics results were shown (**d**) (n=4). **e** MitoSOX staining assay of HepG2-WT and HepG2-AKO by flow cytometry (n=4). Data are analyzed by unpaired two-tailed student’s t test and represented as mean ± SEM. * p<0.05, ** p<0.01, *** p<0.001, **** P<0.0001.


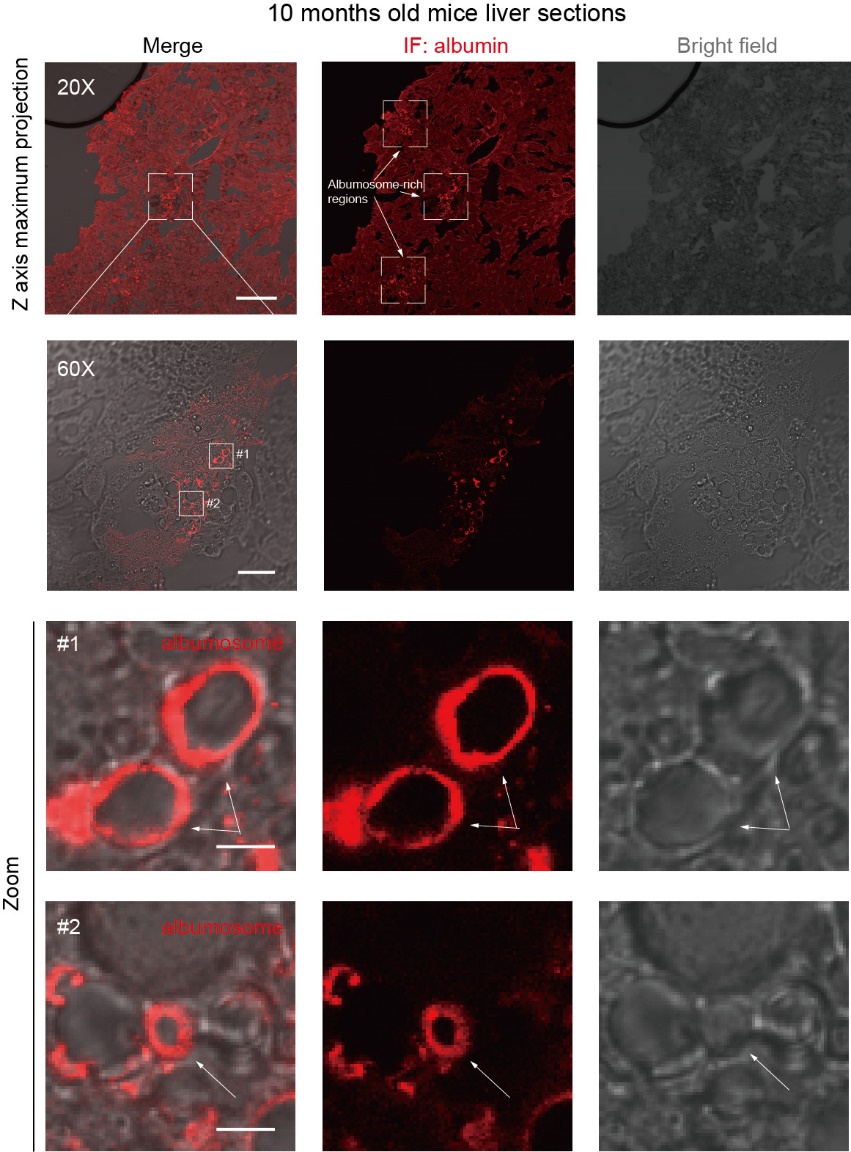


**Figure S3. IF of albumosomes in mice liver sections.**

Liver sections of 10-month-old mice were used in IF. Representative images of maximum brightness projection of multilayers based on z-axis from confocal microscopy by 20X objective. Scale bar: 100μm. Monolayer confocal images by 60X objective. Scale bar: 30μm. Zoomed images regions of #1-#2. Scale bars: 4μm.


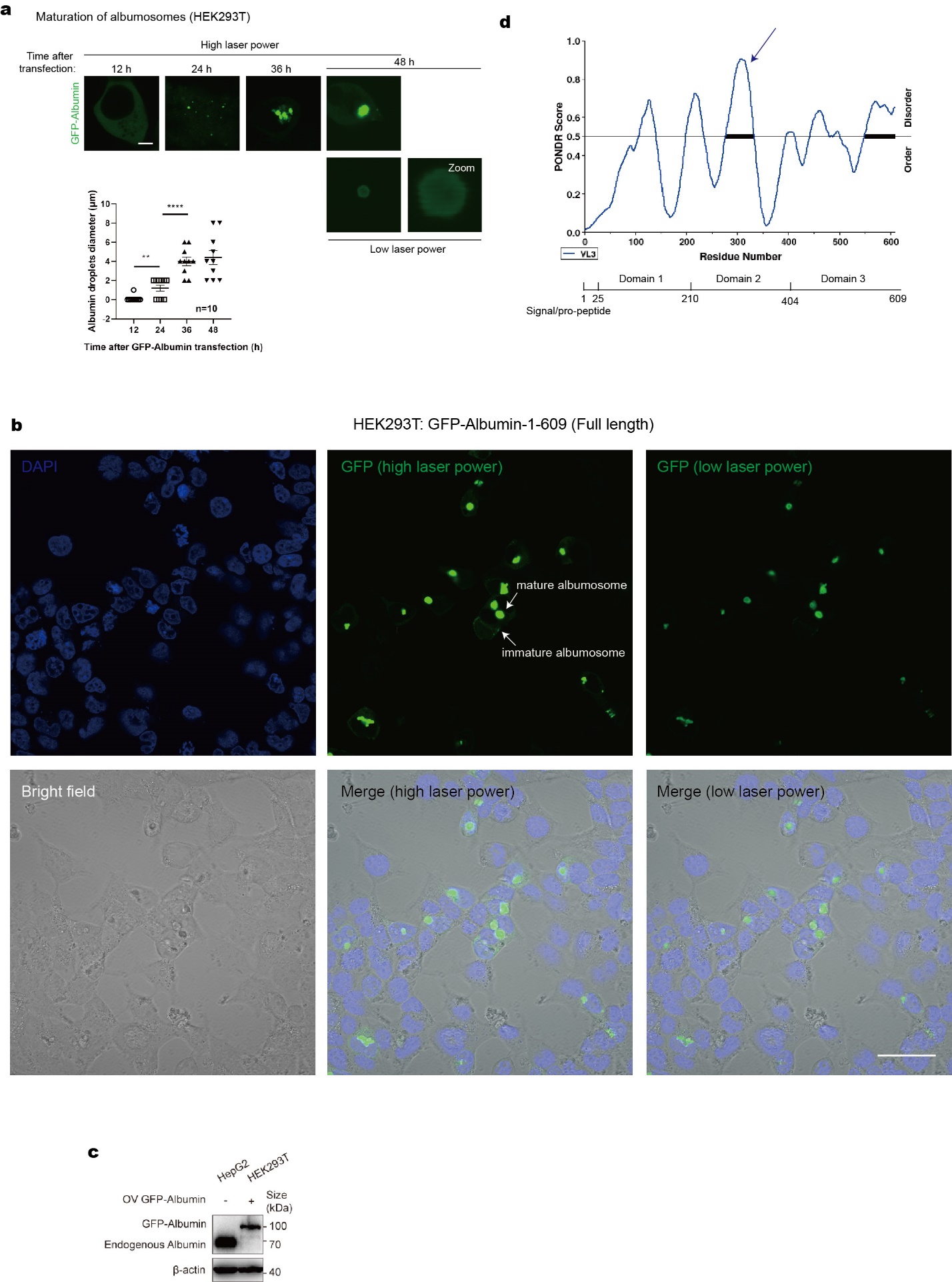


**Figure S4. Supplementary identities of albumosomes.**

**a** Forming process of albumosomes in HEK293T. Confocal microscopy of HEK293T transfected with GFP-Albumin for 12, 24, 36 and 48h. Measurement of the diameter of albumin droplets at different time points. Representative images were shown. Scale bars: 5μm. **b** A wider range of view of albumosomes in HEK293T transfected with GFP-Albumin. GFP-Albumin was transfected into HEK293T for 48h. Then the cells were fixed and stained with DAPI and used in confocal microscopy. Scale bar: 40μm. **c** Westernblot results of the albumin in HepG2 (endogenous albumin) and HEK293T transfected with GFP-Albumin. GFP-Albumin was transfected into HEK293T for 48h before the cells were harvested. **d** Predicted disordered score of human albumin by http://original.disprot.org/metapredictor.php. Data are analyzed by unpaired two-tailed student’s t test and represented as mean ± SEM. ** p<0.01, **** p<0.0001.


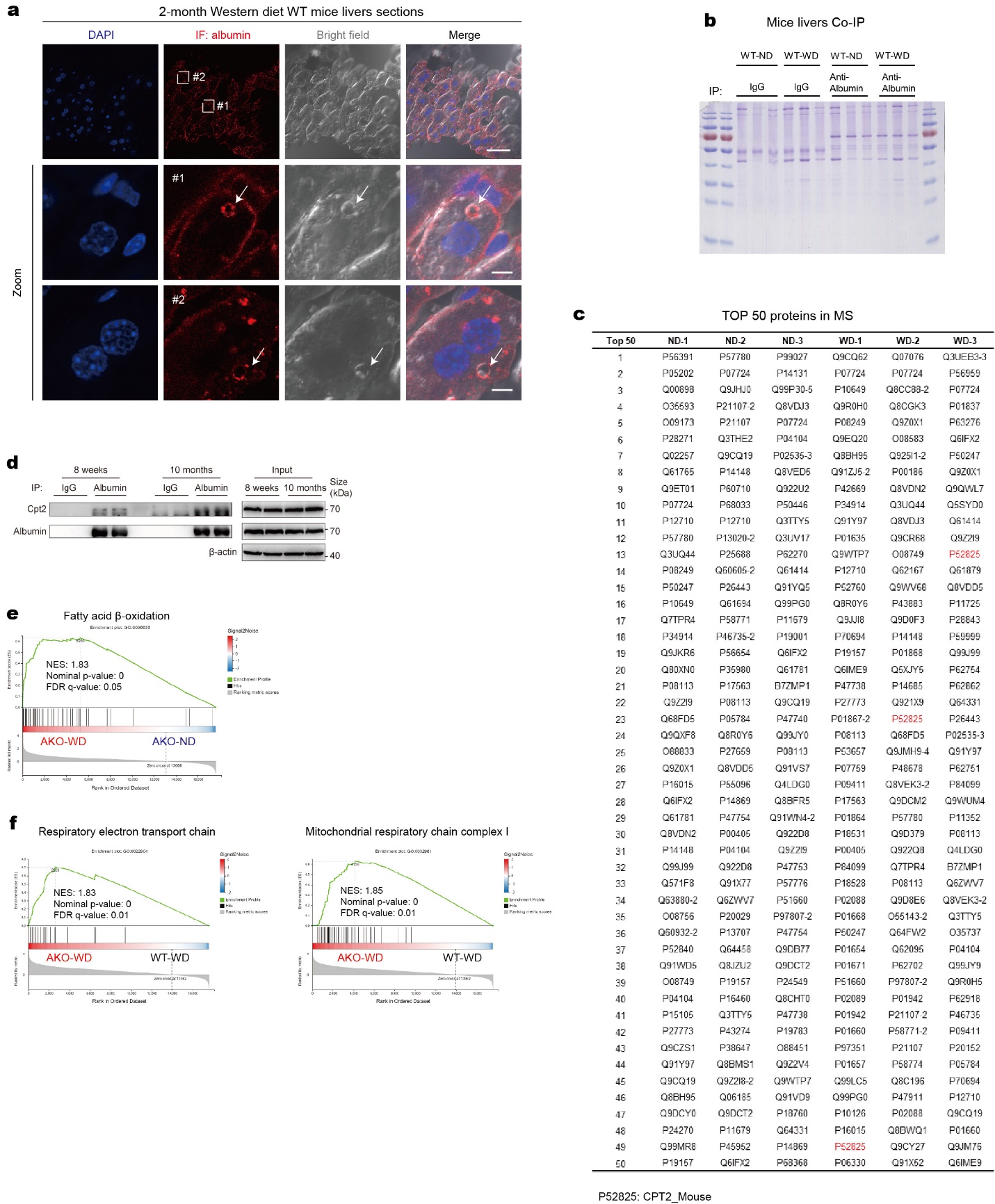


**Figure S5. IF, Co-IP-MS and RNA-Seq results in mice livers.**

**a** IF of albumin in liver sections from two-month WT-WD mice. Representative images were shown. Scale bars: 40μm in initial images and 5μm in zoomed images. **b** Liver tissues from WT-ND and WT-WD were used for Co-IP combined with MS to investigate the interacting proteins of albumin intracellularly. SDS-PAGE and coomassie blue staining of the immunoprecipitated samples were shown (n=3). **c** Top 50 proteins of each group in the results of MS. **d** Co-IP results to verify the interaction between albumin and Cpt2 in mice livers at the age of 8 weeks and 10 months (n=2). **e** GSEA analysis of fatty acid β-oxidation between AKO-WD and AKO-ND livers (n=6). **f** GSEA analysis of respiratory electron transport chain and mitochondrial respiratory chain complex I between AKO-WD and WT-WD livers (n=6).


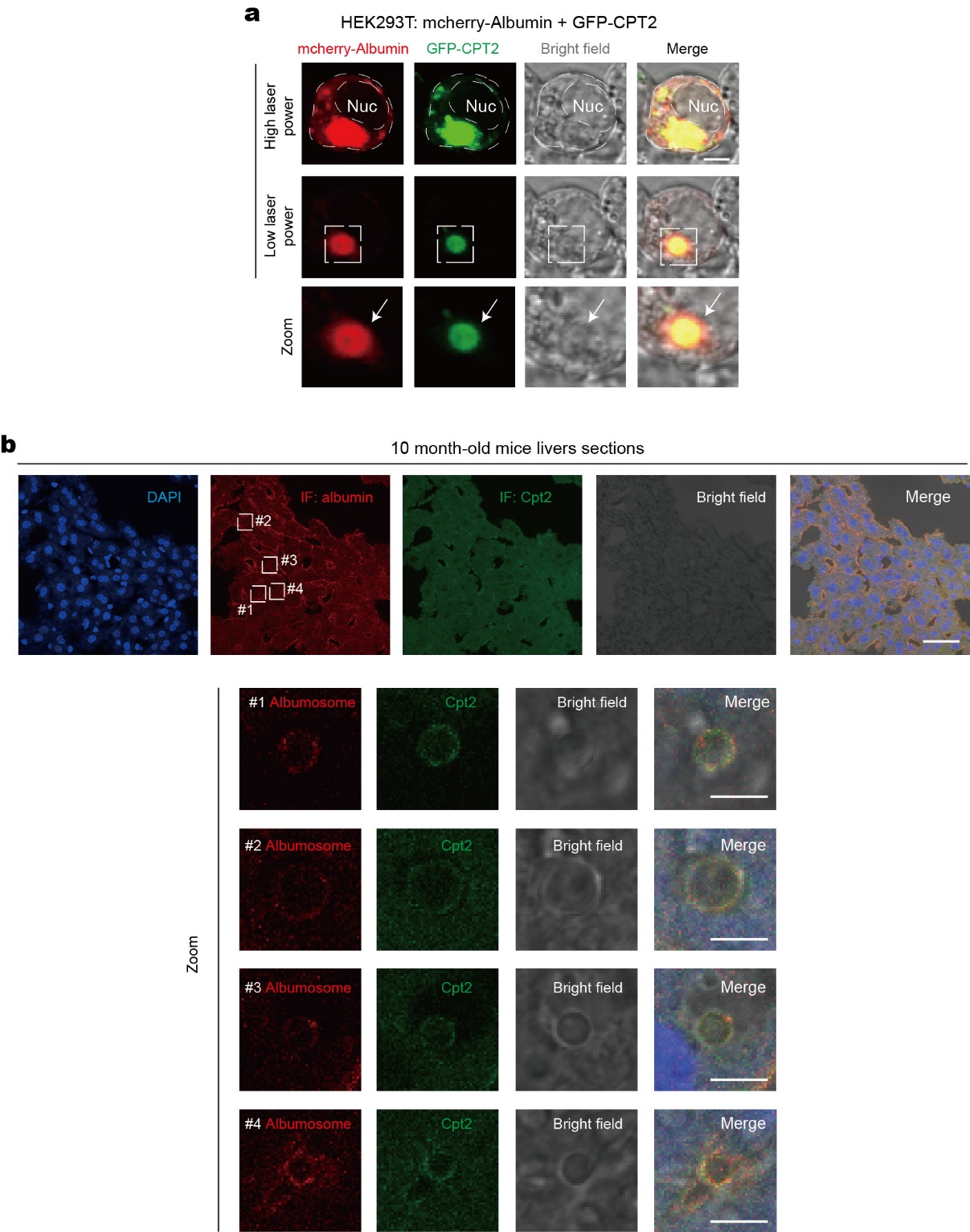


**Figure S6. Albumosomes interact with CPT2 in HEK293T and mice liver sections.**

**a** Confocal microscopy of HEK293T transfected with mcherry-Albumin and GFP-CPT2. Representative images were shown. Scale bar: 5μm. **b** 10-month-old mice liver sections were used in IF to show the co-localization of albumosomes and Cpt2 in mice livers. Representative images were shown. Scale bars: 40μm in initial images and 5μm in zoomed images.


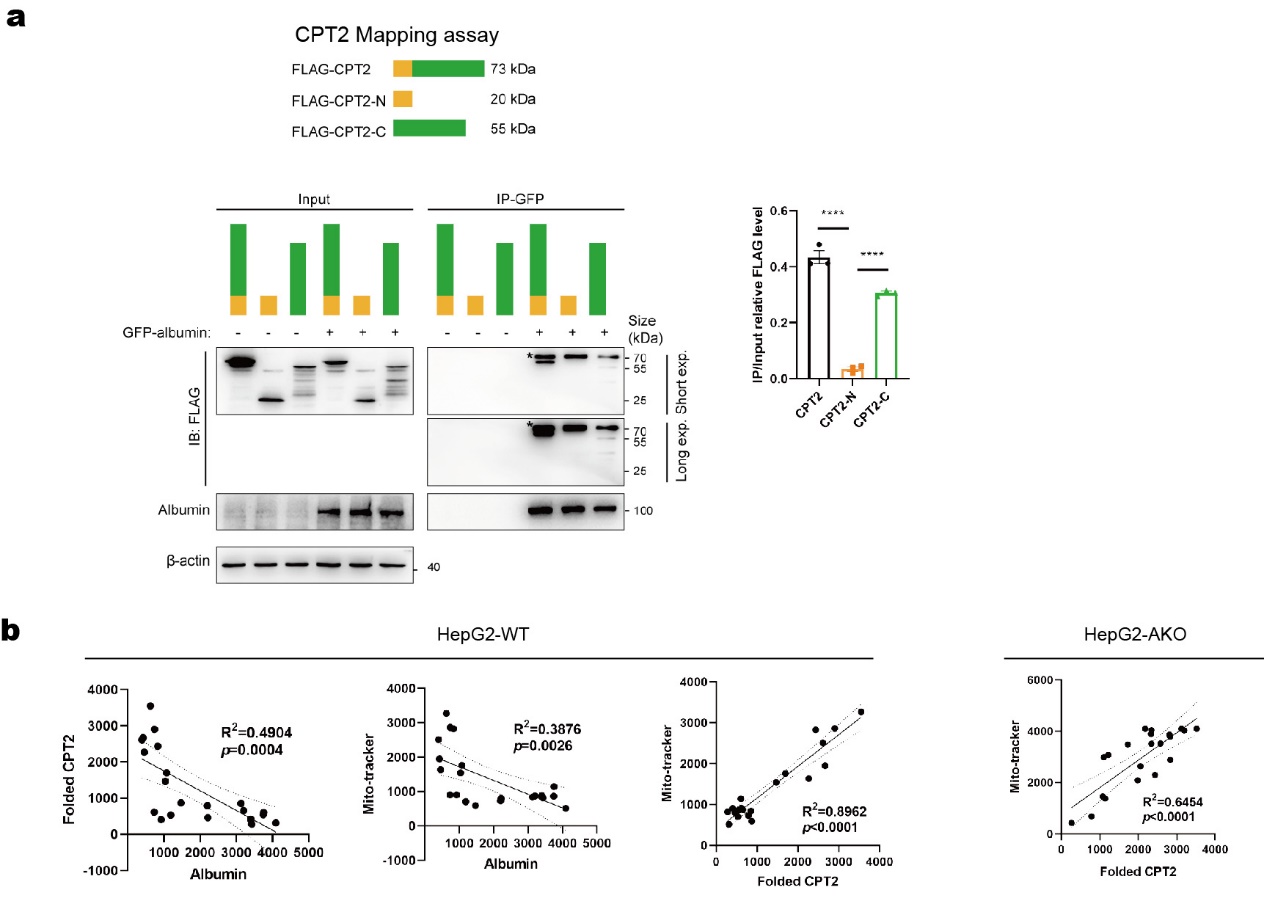


**Figure S7. Albumosomes interact with pre-folding CPT2.**

**a** Mapping of CPT2 and the interaction with albumin and CPT2 fragments in HEK293T cells. FLAG tagged CPT2 fragments were transfected respectively with GFP-Albumin in HEK293T cells. Co-IP assay was used to test the interactions. * unspecific band. **b** Correlation of the intensity of albumin, mitochondrial folded CPT2, and mito-tracker in HepG2-WT and HepG2-AKO for Fig. 5f. Data are analyzed by unpaired two-tailed student’s t test and represented as mean ± SEM. ns. represents no significance. **** P<0.0001.


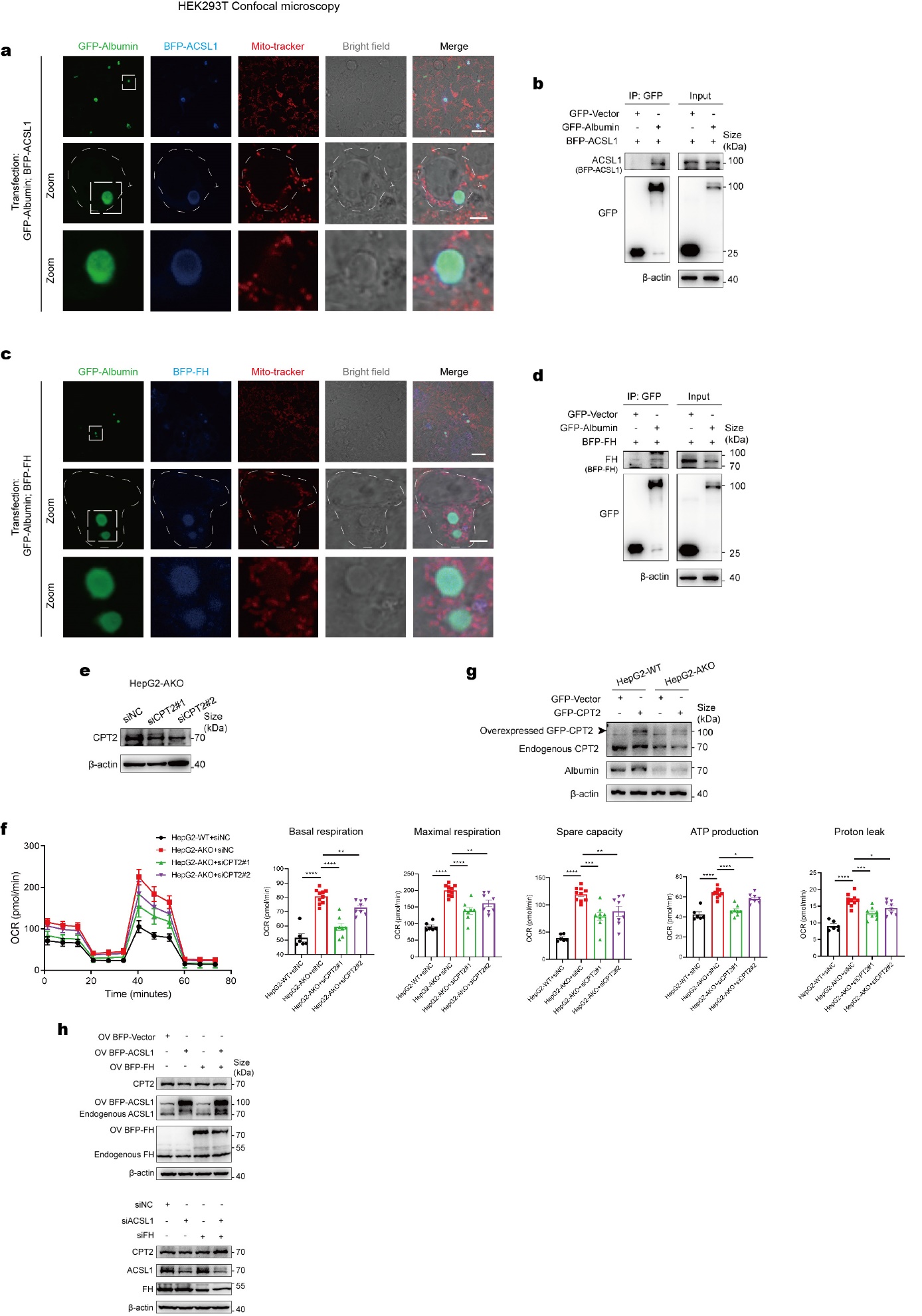


**Figure S8. Albumosomes interact with ACSL1 and FH.**

**a** Confocal microscopy of HEK293T transfected with GFP-Albumin and BFP-ACSL1. Representative images were shown. Scale bars: 30μm in initial images and 5μm in zoomed images. **b** Westernblot validation of the interaction between albumin and ACSL1. Representative images were shown. **c** Confocal microscopy of HEK293T transfected with GFP-Albumin and BFP-FH. Representative images were shown. Scale bars: 30μm in initial images and 5μm in zoomed images. **d** Westernblot validation of the interaction between albumin and FH. Representative images were shown. **e** Westernblot validation of CPT2 silencing by siRNA in HepG2-AKO. **f** Seahorse mitochondrial respiratory assay of HepG2-AKO transfected with CPT2 siRNA or siNC and HepG2-WT transfected with siNC. Basal respiration, maximal respiration, spare capacity, ATP production, and proton leak were analyzed. **g** Westernblot validation of CPT2 overexpression in HepG2-WT and HepG2-AKO. **h** Westernblot of CPT2 under overexpression or knockdown of ACSL1, or FH, or both in HEK293T. Data are analyzed by unpaired two-tailed student’s t test and represented as mean ± SEM. * p<0.05, ** p<0.01, *** p<0.001, **** P<0.0001.


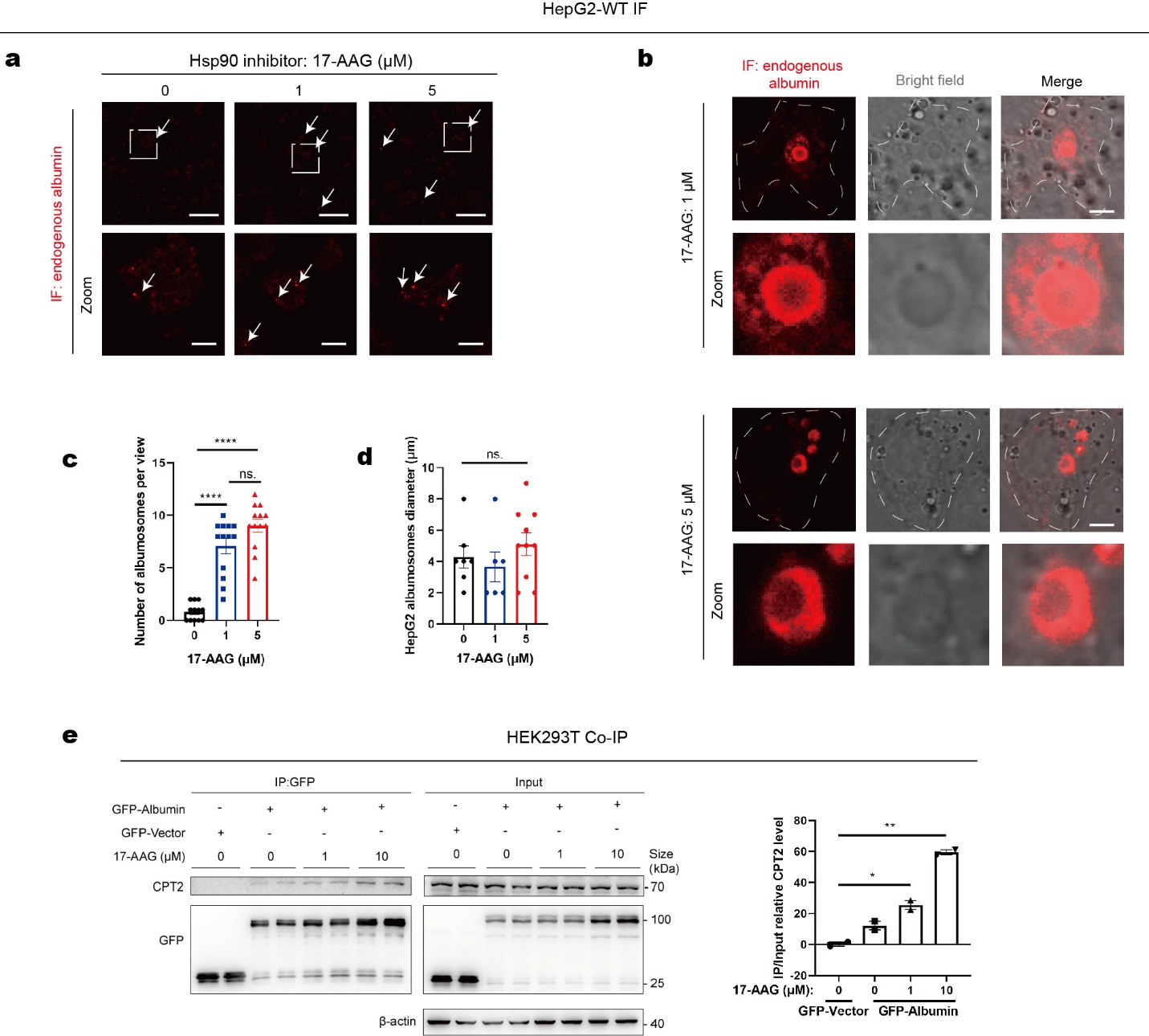


**Figure S9. Hsp90i 17-AAG promotes albumosomes accumulation in HepG2.**

**a** IF of albumin in HepG2-WT after Hsp90i 17-AAG treatment. Representative images were shown. Scale bars: 300μm in initial images and 50μm in zoomed images. **b** Zoomed images of IF to show albumosomes in HepG2-WT treated with Hsp90i 17-AAG. Representative images were shown. Scale bars: 5μm. **c** Number of albumosomes per view in (**a**) (n=12-13).

**d** Diameter of albumosomes in (**a**) (n=6-10). **e** Co-IP assay to show the interaction between GFP-Albumin and CPT2 in HEK293T treated with Hsp90i 17-AAG (n=2). Data are analyzed by unpaired two-tailed student’s t test and represented as mean ± SEM. ns. represents no significance. * p<0.05, ** p<0.01, **** P<0.0001.


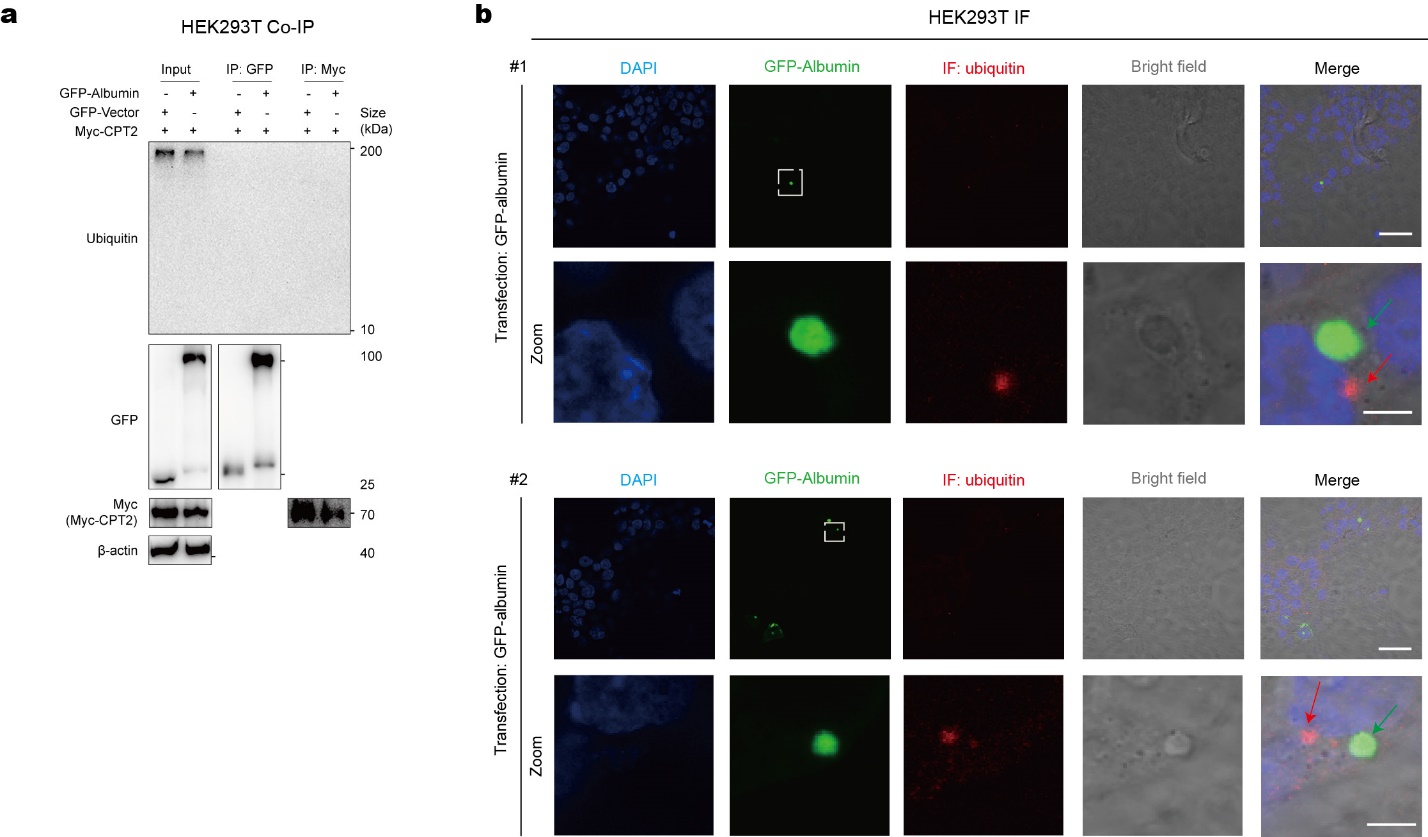


**Figure S10. Albumosomes and albumosomal trapped CPT2 have no ubiquitination.**

**a** Co-IP of GFP and Myc tags in HEK293T transfected with GFP-Albumin and Myc-CPT2. Westernblot results of ubiquitin. Representative images were shown. **b** IF of ubiquitin in HEK293T transfected with GFP-Albumin. Representative images were shown. Scale bars: 40μm in initial images and 5μm in zoomed images.


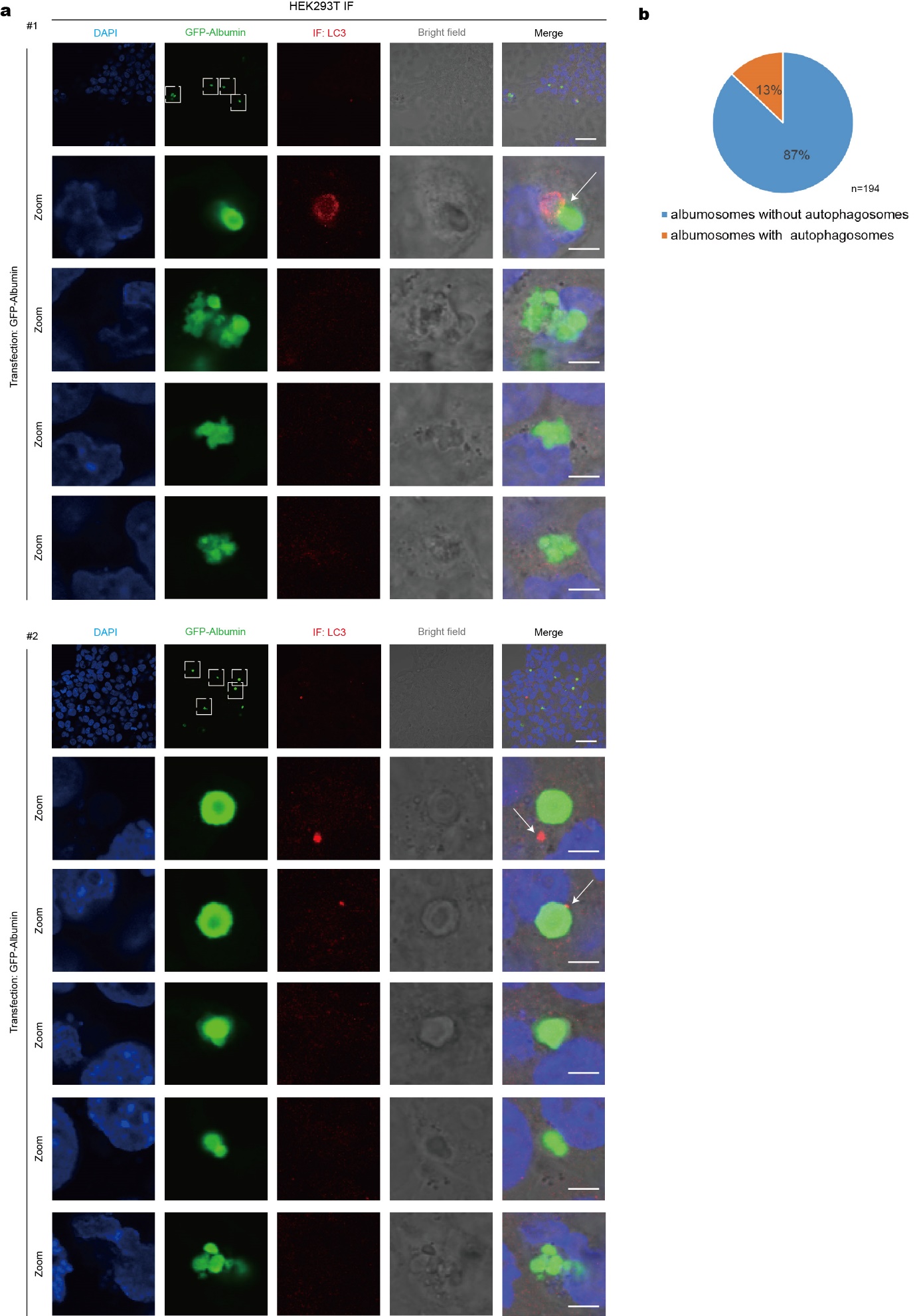


**Figure S11. Albumosomes have little interaction with autophagosomes.**

**a** IF of LC3 in HEK293T transfected with GFP-Albumin. Representative images were shown. White arrows indicate the potential interactions between albumosomes and autophagosomes. Scale bars: 40μm in initial images and 5μm in zoomed images. **b** Statistical results of the ratio of albumosomes with or without autophagosomes around them.


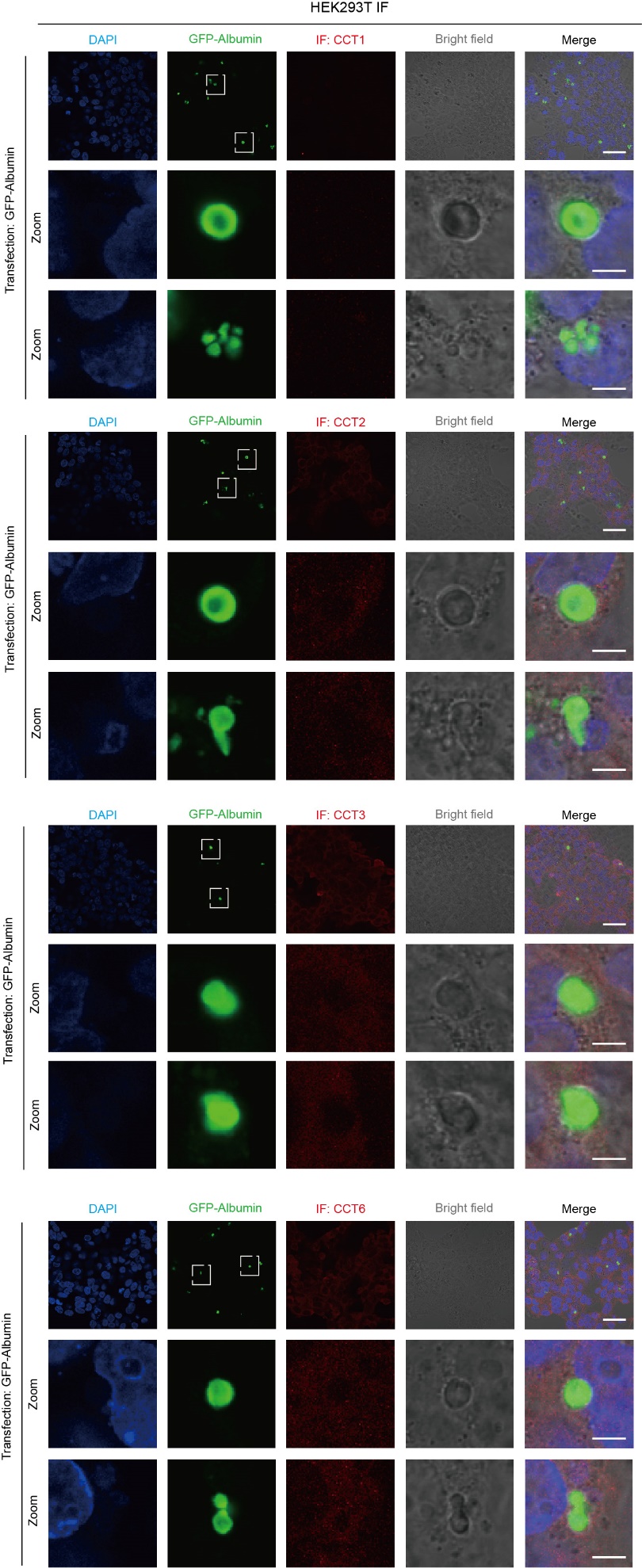


**Figure S12. Albumosomes have little interaction with CCT family proteins.**

IF of CCT1, CCT2, CCT3, CCT6 in HEK293T transfected with GFP-Albumin. Representative images were shown. Scale bars: 40μm in initial images and 5μm in zoomed images.


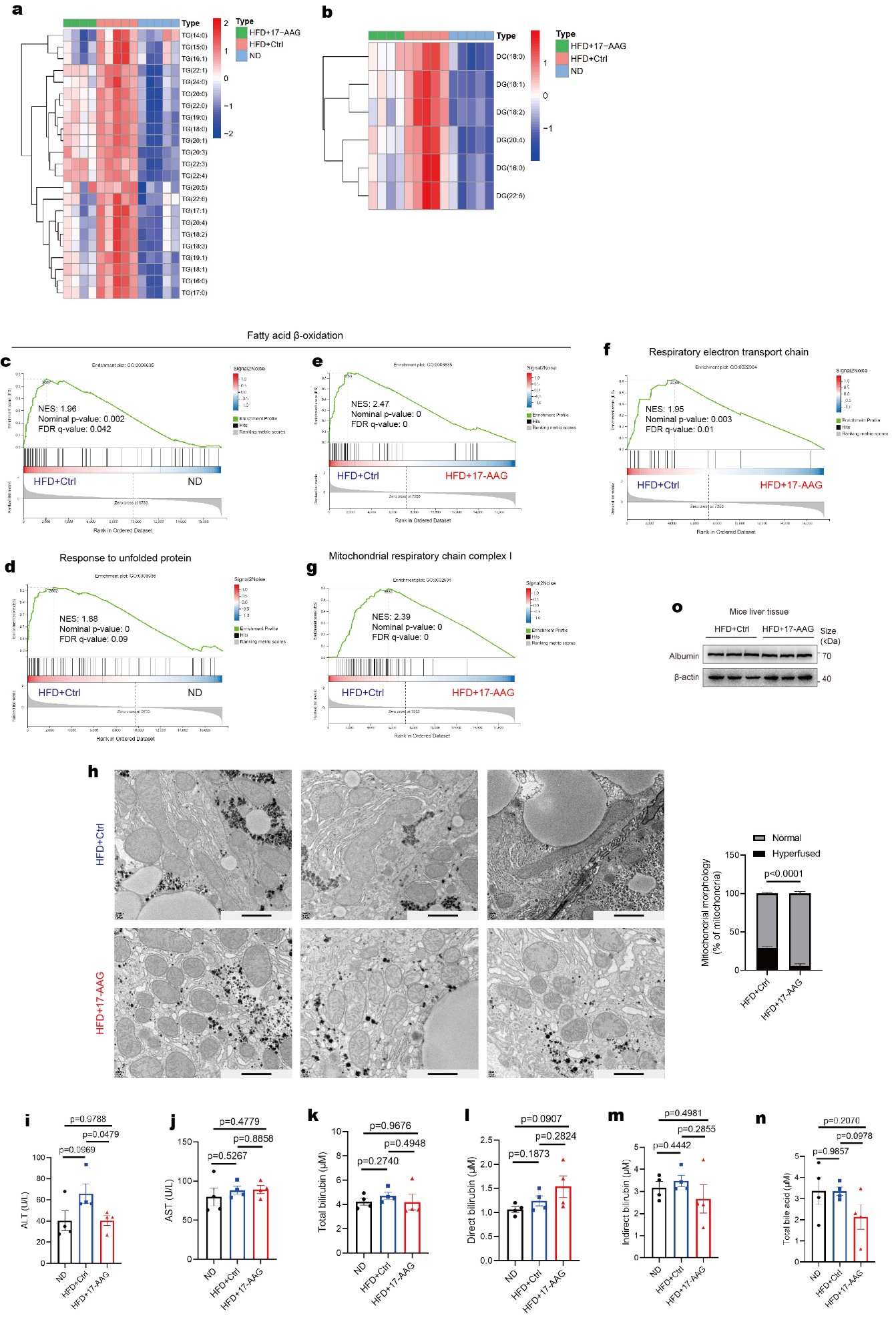


**Figure S13. Lipidomics, RNA-Seq, and TEM of livers and liver function tests.**

**a**-**b** Triglyceride (**a**) and diglyceride (**b**) in the lipidomics of mice liver in HFD+17-AAG, HFD+Ctrl, and ND groups. Data were shown as heatmaps (n=4-5). **c**-**g** GSEA analysis of fatty acid β-oxidation, response to unfolded protein, respiratory electron transport chain, and mitochondrial respiratory chain complex I in HFD+17-AAG, HFD+Ctrl, and ND groups (n=4-5). **h** TEM of liver sections of HFD+Ctrl and HFD+17-AAG mice. The hyperfused level of mitochondria was analyzed (n=12-13). Representative images were shown. Scale bars: 1mm. **i**-**n** Liver function tests of the serum of HFD+17-AAG, HFD+Ctrl, and ND groups (n=4-5). **o** Westernblot results to show albumin in the liver tissues of HFD+17-AAG and HFD+Ctrl (n=3). Data are analyzed by unpaired two-tailed student’s t test and represented as mean ± SEM.


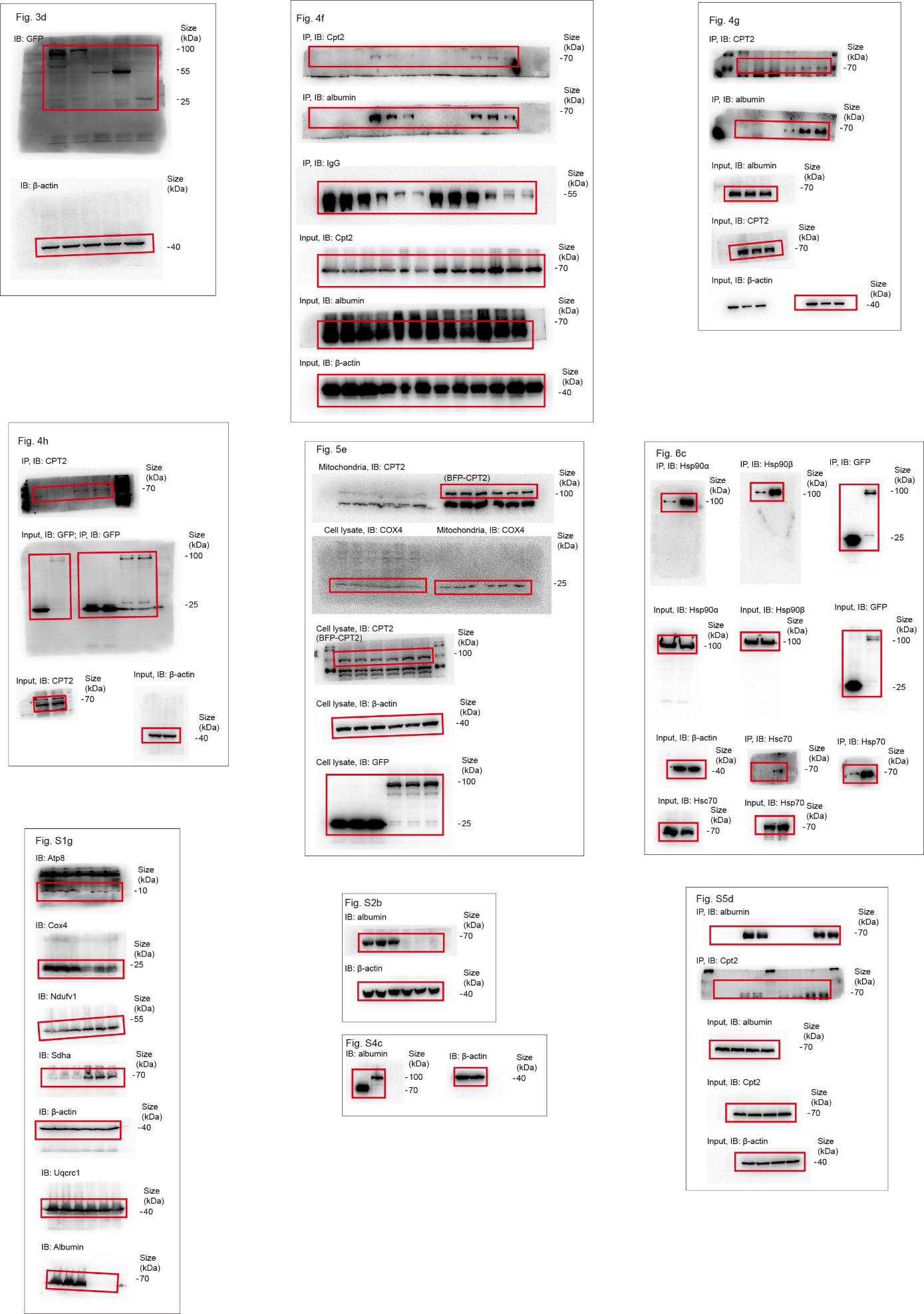


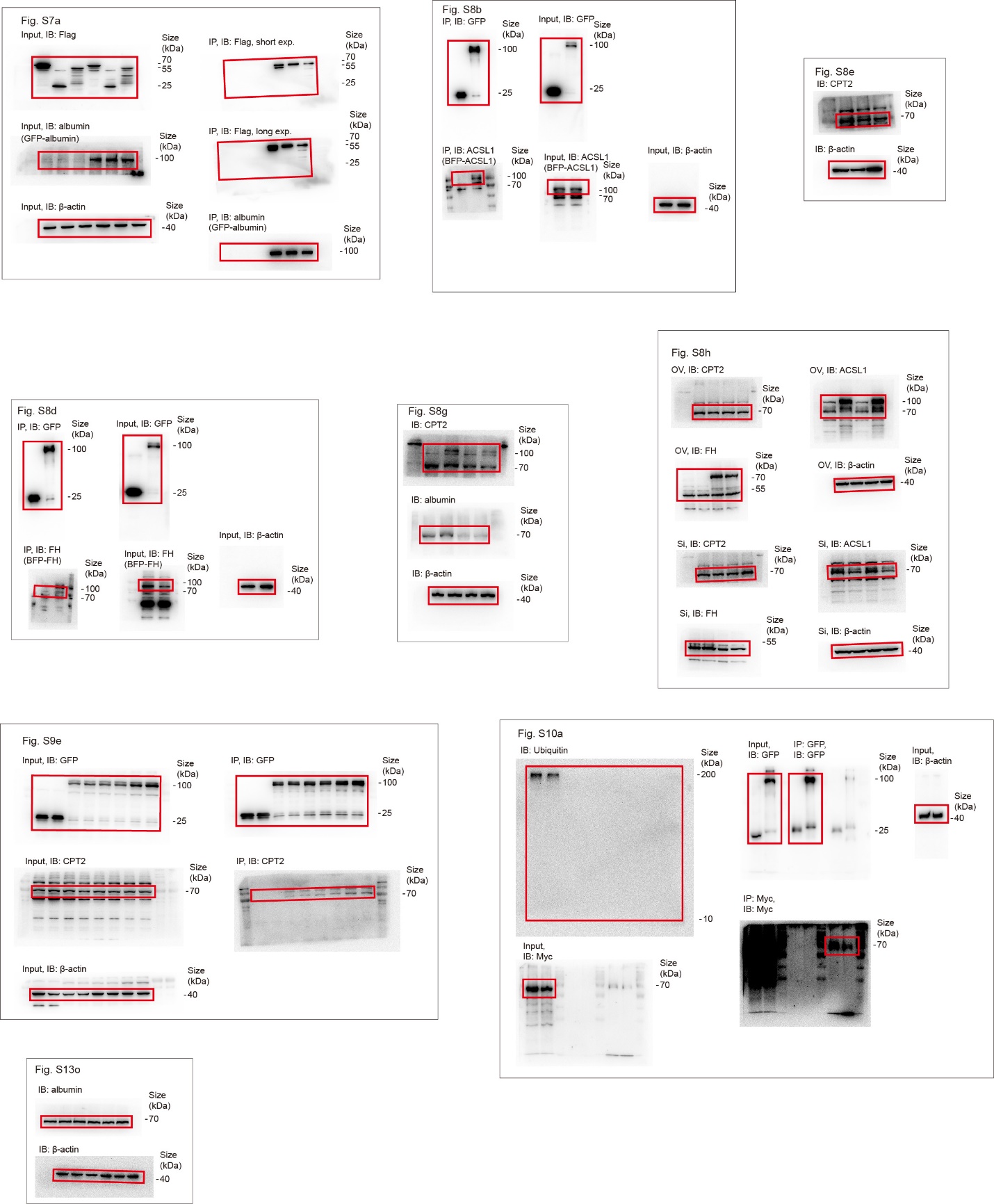

Supplement: Supplementary file 1 — Supplementary materials [file 41392_2023_1437_MOESM1_ESM.docx]
